# Towards defining the chloroviruses: a genomic journey through a genus of large DNA viruses

**DOI:** 10.1186/1471-2164-14-158

**Published:** 2013-03-08

**Authors:** Adrien Jeanniard, David D Dunigan, James R Gurnon, Irina V Agarkova, Ming Kang, Jason Vitek, Garry Duncan, O William McClung, Megan Larsen, Jean-Michel Claverie, James L Van Etten, Guillaume Blanc

**Affiliations:** 1 Information Génomique & Structurale, IGS UMR7256, CNRS, Aix-Marseille Université, Marseille, FR-13288, France; 2 Department of Plant Pathology, University of Nebraska, Lincoln, NE, 68583-0722, USA; 3 Nebraska Center for Virology, University of Nebraska, Lincoln, NE, 68583-0900, USA; 4 Biology Department, Nebraska Wesleyan University, Lincoln, NE, 68504, USA; 5 Current address: Department of Biology, Indiana University, Bloomington, IN, 47408, USA

## Abstract

**Background:**

Giant viruses in the genus *Chlorovirus* (family *Phycodnaviridae*) infect eukaryotic green microalgae. The prototype member of the genus, *Paramecium bursaria* chlorella virus 1, was sequenced more than 15 years ago, and to date there are only 6 fully sequenced chloroviruses in public databases. Presented here are the draft genome sequences of 35 additional chloroviruses (287 – 348 Kb/319 – 381 predicted protein encoding genes) collected across the globe; they infect one of three different green algal species. These new data allowed us to analyze the genomic landscape of 41 chloroviruses, which revealed some remarkable features about these viruses.

**Results:**

Genome colinearity, nucleotide conservation and phylogenetic affinity were limited to chloroviruses infecting the same host, confirming the validity of the three previously known subgenera. Clues for the existence of a fourth new subgenus indicate that the boundaries of chlorovirus diversity are not completely determined. Comparison of the chlorovirus phylogeny with that of the algal hosts indicates that chloroviruses have changed hosts in their evolutionary history. Reconstruction of the ancestral genome suggests that the last common chlorovirus ancestor had a slightly more diverse protein repertoire than modern chloroviruses. However, more than half of the defined chlorovirus gene families have a potential recent origin (after Chlorovirus divergence), among which a portion shows compositional evidence for horizontal gene transfer. Only a few of the putative acquired proteins had close homologs in databases raising the question of the true donor organism(s). Phylogenomic analysis identified only seven proteins whose genes were potentially exchanged between the algal host and the chloroviruses.

**Conclusion:**

The present evaluation of the genomic evolution pattern suggests that chloroviruses differ from that described in the related *Poxviridae* and *Mimiviridae*. Our study shows that the fixation of algal host genes has been anecdotal in the evolutionary history of chloroviruses. We finally discuss the incongruence between compositional evidence of horizontal gene transfer and lack of close relative sequences in the databases, which suggests that the recently acquired genes originate from a still largely un-sequenced reservoir of genomes, possibly other unknown viruses that infect the same hosts.

## Background

Viruses in the family *Phycodnaviridae*, together with those in the *Poxviridae*, *Iridoviridae*, *Ascoviridae*, *Asfarviridae* and the *Mimiviridae* are believed to have a common evolutionary ancestor and are referred to as nucleocytoplasmic large DNA viruses (NCLDV) [1-3]. Members of the *Phycodnaviridae* consist of a genetically diverse, but morphologically similar, group of large dsDNA-containing viruses (160 to 560 kb) that infect eukaryotic algae [4,5]. These large viruses are found in aquatic environments, from both terrestrial and marine waters throughout the world. They are thought to play dynamic, albeit largely undocumented roles in regulating algal communities, such as the termination of massive algal blooms [6-8], which has implications in global geochemical cycling and weather patterns [9].

Currently, the phycodnaviruses are grouped into 6 genera, initially based on host range and subsequently supported by sequence comparison of their DNA polymerases [10]. Members of the genus *Chlorovirus* infect chlorella-like green algae from terrestrial waters, whereas members of the other five genera (*Coccolithovirus*, *Phaeovirus*, *Prasinovirus*, *Prymnesiovirus* and *Raphidovirus*) infect marine green and brown algae. Currently, 24 genomes of members in four phycodnavirus genera are present in Genbank. Comparative analysis of some of these genomes has revealed more than 1000 unique genes with only 14 genes in common among the four genera [4]. Thus gene diversity in the phycodnaviruses is enormous.

Here we focus on phycodnaviruses belonging to the genus *Chlorovirus*, referred to as chloroviruses (CV). These viruses infect certain unicellular, eukaryotic, ex-symbiotic chlorella-like green algae, which are often called zoochlorellae; they are associated with either the protozoan *Paramecium bursaria*, the coelenterate *Hydra viridis* or the heliozoon *Acanthocystis turfacea*[11]. Three such zoochlorellae are *Chlorella* NC64A, recently renamed *Chlorella variabilis*[12], *Chlorella* SAG 3.83 (renamed *Chlorella heliozoae*) and *Chlorella* Pbi (renamed *Micratinium conductrix*). Viruses infecting these three zoochlorellae will be referred to as NC64A-, SAG-, or Pbi-viruses.

Since the initial sequencing of the prototype CV, *Paramecium bursaria* chlorella virus 1 [13,14], more than 15 years ago, only 5 more whole-genome sequences of CVs have been reported [15-17]. These 6 sequences reveal many features that distinguish them from other NCLDV including genes encoding a translation elongation factor EF-3, enzymes required to glycosylate proteins [18], enzymes required to synthesize the polysaccharides hyaluronan and chitin, polyamine biosynthetic enzymes, proteins that are ion transporters and ones that form ion channels including a virus-encoded K^+^ channel (designated Kcv) [19], a SET domain-containing protein (referred to as vSET) that dimethylates Lys^27^ in histone 3 [20,21], and many DNA methyltransferases and DNA site-specific endonucleases [22,23]. Moreover, the evolution of large DNA viruses is subject to intense debate. Questions include, how did this vast gene diversity arise? Are genes captured from organisms or viruses, or did genome reduction occur from a larger ancestor? Here we address these questions by sequencing and comparing the genomes of 41 CVs infecting 3 different green algal species.

## Results and discussion

**Figure 1:**
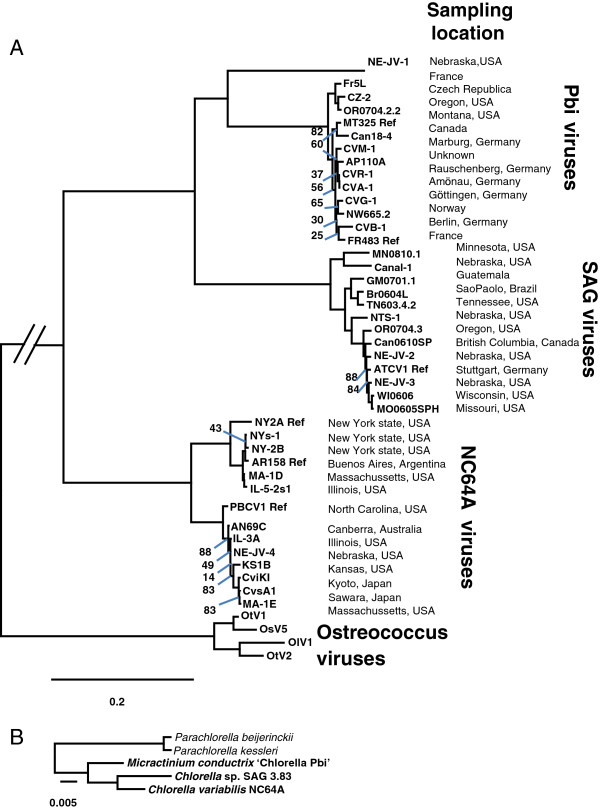
**Phylogenies of chloroviruses and algal hosts. A**: ML tree of chloroviruses based on a concatenated alignment of 32 core protein families (7762 gap-free sites). The phylogenetic tree was computed using the WAG + G + I substitution model. Branch support was estimated from 1000 bootstrap replicates. We only show bootstrap values < 90%. Ostreococcus viruses serve as an outgroup to root the tree. **B**: ML tree of algal hosts based on 18S RNA alignment (2266 gap-free sites). The phylogenetic tree was computed using the GTR + G + I substitution model. All interior branches received maximal support (100%). *Parachlorella* spp. are used as outgroup.

Terrestrial water samples have been collected throughout the world over the past 25 years and plaque-assayed for CVs. The viruses selected for sequencing (Figure 1) were chosen from a collection of more than 400 isolates with the intention of evaluating various phenotypic characteristics and geographic origins as indicators of diversity; an equal number of isolates infecting each of the three hosts were selected. However, this selection of viruses does not represent a biogeographic survey.

The viral genomes were assembled into 1 to 39 large contigs (with an average length of 40 Kb), had cumulated sizes ranging from 287 to 348 Kb and an average read coverage between 27 and 107 (Table 1). Contig extremities often contained repeated sequences that interfered with the assembly process and precluding obtaining a single chromosome contig. Two virus assemblies contained a large number of contigs – i.e., Fr5L and MA-1E containing 22 and 39 contigs respectively. In fact, >90% of the Fr5L and MA-1E sequences were contained in 5 and 9 large contigs, respectively, which is similar to the number of large contigs in the other virus assemblies. The remaining contigs were small (<1 kb for the majority) and showed strong sequence similarity with reference genomes, which suggests that they did not arise from contamination. Like the previously sequenced CVs, the G + C content of the newly sequenced genomes was between 40% and 52%. Moreover, the G + C content was highly homogeneous and specific among viruses infecting the same host: i.e., NC64A, Pbi and SAG viruses had a median G + C content of 40%, 45% and 49%, respectively with low standard deviation in each group (<0.14%).

**Table 1:** **General features of the sequenced *chlorovirus *genomes**

**Virus**	**Host**	**# Contigs**	**Genome size (Kb)**	**Sequence coverage**	**% GC**	**# protein genes**	**# tRNA genes**	**# protein families**	**Genbank accession number**
AN69C	NC64A	8	332	29x	40	362	10	278	JX997153
CviKI	NC64A	8	308	55x	40	336	14	271	JX997162
CvsA1	NC64A	9	310	36x	40	342	14	272	JX997165
IL-3A	NC64A	3	323	50x	40	349	12	273	JX997169
IL-5-2s1	NC64A	9	344	65x	41	379	8	281	JX997170
KS1B	NC64A	7	287	46x	40	319	13	257	JX997171
MA-1D	NC64A	9	339	45x	41	371	11	288	JX997172
MA-1E	NC64A	39	336	27x	40	376	14	269	JX997173
NE-JV-4	NC64A	8	328	41x	40	352	11	276	JX997179
NY-2B	NC64A	5	344	59x	41	371	8	281	JX997182
NYs-1	NC64A	9	348	64x	41	381	7	286	JX997183
AP110A	Pbi	6	327	27x	44	348	9	269	JX997154
Can18-4	Pbi	11	329	52x	45	357	10	271	JX997157
CVA-1	Pbi	8	326	36x	45	346	9	270	JX997159
CVB-1	Pbi	8	319	90x	44	346	10	272	JX997160
CVG-1	Pbi	7	318	48x	45	333	9	262	JX997161
CVM-1	Pbi	5	327	48x	44	341	9	268	JX997163
CVR-1	Pbi	11	329	39x	45	351	9	268	JX997164
CZ-2	Pbi	11	305	39x	45	340	10	262	JX997166
Fr5L	Pbi	22	302	58x	45	345	11	257	JX997167
NE-JV-1	Pbi	8	326	45x	47	337	3	265	JX997176
NW665.2	Pbi	6	325	62x	44	350	8	263	JX997181
OR0704.2.2	Pbi	8	313	53x	45	344	7	261	JX997184
Br0604L	SAG	2	295	65x	49	346	9	272	JX997155
Can0610SP	SAG	1	307	61x	49	341	13	267	JX997156
Canal-1	SAG	4	293	50x	51	336	10	277	JX997158
GM0701.1	SAG	4	315	71x	48	362	10	272	JX997168
MN0810.1	SAG	6	327	57x	52	343	9	268	JX997174
MO0605SPH	SAG	3	289	107x	49	323	11	271	JX997175
NE-JV-2	SAG	4	319	40x	48	346	13	271	JX997177
NE-JV-3	SAG	3	298	63x	49	334	12	268	JX997178
NTS-1	SAG	4	323	35x	48	364	7	271	JX997180
OR0704.3	SAG	5	311	49x	49	342	13	272	JX997185
TN603.4.2	SAG	3	321	28x	49	351	9	276	JX997186
WI0606	SAG	7	289	58x	50	329	11	271	JX997187

Gene prediction algorithms identified 319 to 381 protein-encoding genes (CDSs) in each genome, of which 48% were given a functional annotation. Furthermore, each genome was predicted to contain between 5 and 16 tRNA genes. These features resemble the 6 previously sequenced CV genomes that had 329 to 416 protein-encoding genes and 7 to 11 tRNA genes [14-16]. However, we cannot rule out the possibility that a small number of genes may be missing if their location coincides with the gaps in the CV genome assemblies. We attempted to complete the assembly of 6 of the incompletely assembled viruses by PCR-sequencing across gaps. However, in many cases, repetitive sequences in adjacent contigs made it difficult to synthesize suitable primers. Since we had >20X depth of coverage in non-repetitive regions, we suspect that the gaps were actually sequenced during the genomic sequencing phase of the project but that the assembly software discarded reads containing repetitive sequences that it was unable to confidently align with sequences at the ends of contigs. Nonetheless, we successfully sequenced 16 of 28 gaps among the 6 viruses and the gap sizes ranged from 1 to 634 nts. Thus the gaps are predicted to be very small.

### Core and host-specific proteins in CVs

Predicted CV proteins were organized into 531 clusters of two or more orthologous proteins plus 101 singleton CV proteins (Additional file 1: Table S1). The largest protein family contained 429 members, which were similar to intron-encoded endonucleases.

The core protein family set consisted of 155 protein families shared by all the CVs, which represent 56% of the average protein family content of CVs; the majority (66%) of those proteins have an annotated function. Thirty-eight core protein families were also ubiquitous in four *Ostreococcus* viruses [24-27], which are members of the genus *Prasinovirus* that are closely related to the chloroviruses; these core proteins include the NCLDVs hallmark genes (DNA polymerase B, major capsid protein, primase-helicase, packaging ATPase and transcription factor TFII) [2]. The remaining 117 CV core protein families grouped into a variety of functions, with a preponderance of proteins associated with the virion particle (i.e., capsid proteins), degradation of the host cell-wall (i.e., alginate lyase, chitinase and chitosanase), DNA replication, transcription and protein maturation. These enzymatic functions and structural proteins form the backbone of CV metabolism that enable them to propagate, spread from host to host, enter into the cell, and regulate the cellular machinery to promote virus replication.

In addition, orthologous protein families were identified that are ubiquitous to viruses infecting one of the algal hosts (i.e., NC64A, SAG or Pbi) but absent in all the other CVs. These proteins are presumably involved in the mechanism of host recognition and specificity. The host-specific protein sets were much smaller both in terms of size and number of predicted functions. We identified 11 orthologous clusters specific to NC64A viruses, of which 2 have annotated functions, including an aspartate carbamoyltransferase involved in *de novo* pyrimidine biosynthesis in the plastids of land plants [24], and an homolog to a plant thylakoid formation protein involved in sugar sensing and chloroplast development [25]. This suggests that the adaptation of CVs to the NC64A host might require a more intricate control of the chloroplast and nucleotide biosynthesis by the NC64A viruses. The NC64A viruses have the most biased nucleotide composition of all the CVs (i.e., 40% G + C), which may explain why these viruses require a higher degree of control of the available nucleotide pool. Pbi and SAG viruses had 6 and 9 host -specific core genes, respectively, none of which have known functions, making it difficult to predict the mechanisms underlying host specificity.

Eight protein families had an opposite conservation pattern; they were systematically absent in viruses infecting the same algal host but were present in all the other CVs. Four of them had a predicted function: SAG and NC64A viruses lack an ankyrin repeat domain-containing protein and a glycosyltransferase, respectively. Pbi viruses lack GDP-D-mannose dehydratase and GDP-L-fucose synthase that catalyze two consecutive steps in the biosynthesis of GDP-L-fucose. GDP-L-fucose is the sugar nucleotide intermediate in the synthesis of fucosylated glycolipids, oligosaccharides and glycoproteins [28]. These two enzymes exist in all the other sequenced phycodnaviruses that infect green algae, including *Ostreococcus* viruses, *Micromonas* viruses, and *Bathycoccus* viruses. The long ancestry of GDP-D-mannose dehydratase and GDP-L-fucose synthase suggests that GDP-L-fucose is an important metabolite in the general metabolism of phycodnaviruses that infect green algae. Thus the loss of these two corresponding genes in the Pbi virus lineage may be regarded as a significant evolutionary step that could mark specialization to the host. However, experimental evidence indicates that two sequenced Pbi viruses, MT325 and CVM-1, have fucose as one of the components of their major capsid protein (Tonetti et al., personal communication), indicating that even in the absence of the viral-encoded proteins, Pbi viruses obtain GDP-L-fucose from their host. The loss of the two genes was perhaps made possible by either a greater availability of fucose in the cytoplasm of the Pbi host or a lesser need for fucose by the virus.

The remaining 443 protein clusters had scattered distributions among CVs infecting the three algal hosts. In contrast to the core CV protein set, these protein sets included a significant number of proteins potentially involved in cell-wall glycan metabolism and protein glycosylation, ion channels and transporters, polyamine metabolism, and DNA methytransferases and DNA restriction endonucleases. The different combinations of dispensable genes existing in the CVs are presumably the origin of the phenotypic variations observed between them such as efficiency of infection, burst size, infection dynamics, nature of protein glycans, and genome methylation [11].

### Novel protein genes

One hundred and sixty-six clusters totaling 403 proteins did not have an orthologous member in any of the reference viruses. The corresponding genes are thus seen for the first time in CV and encode potential new functionalities. Only 22 new clusters had a match in a public database, the rest of the proteins were annotated as “hypothetical protein.” Furthermore, only 6 clusters were homologous to proteins annotated with functional attributes (Additional file 2: Table S2). They include a fumarate reductase possibly involved in anaerobic mitochondrial respiration [29], and five proteins with generic functional annotation: acetyltransferase, SAM-dependent methyltransferases, nitroreductase, glycosyl hydrolase and helicase.

### Phylogeny

Phylogenetic relationships between the sequenced CVs and *Ostreococcus* viruses were determined from an analysis of the concatenated alignment of 32 protein families encoded by a single gene in each genome. *Ostreococcus* viruses were treated as an outgroup to root the phylogenetic trees. These genes represent a subset of the “core” CV genes and are mostly involved in basic replication processes. The resulting maximum likelihood (ML) phylogenetic tree is shown in Figure 1A. All branches are associated with high bootstrap values (>90%) except for those containing very similar viruses, for which the exact timing/order of separation events could not be resolved unambiguously. Phylogenetic trees were also inferred by Neighbor Joining (NJ) and Maximum Parsimony (MP) methods using the same sequence dataset (Additional file 3: Figure S1 and Additional file 4: Figure S2). The MP tree had a topology identical to the ML tree while the NJ tree differed by 5 branches associated with low bootstrap values in both the ML and MP trees. In addition, a ML phylogenetic tree of the algal hosts was reconstructed (Figure 1B) from their 18S RNA sequences using *Parachlorella* species as the outgroup on the basis of a previous phylogenetic study of *Chlorellaceae*[12].

The phylogeny study revealed three important features about CV evolution. First, although the CVs were isolated from diverse locations across 5 continents, the phylogenetic trees show that viruses infecting the same algal host always clustered in monophyletic clades. This suggests that the most recent common ancestor of each virus subgenus already infected the same algal host lineage as today’s representatives and that the evolutionary events that led viruses to adapt and specialize to a given host occurred only once in their history. Second, the branching pattern of the three main virus clades does not follow the phylogeny of their algal hosts, which rules out the simplest co-evolution scenario whereby the algae and virus lineages separated in concert. Instead, the phylogenetic evidence strongly suggests that the CVs have changed hosts at least once in their evolutionary history. Finally, while most of the newly sequenced CVs are a close relative of previously sequenced CVs, the basal and isolated phylogenetic position of virus NE-JV-1 within the Pbi virus clade make it the first representative of a new subgroup of CVs that was previously unknown. NE-JV-1 only shares 73.7% amino acid identity on average with the other Pbi viruses in the 32 core proteins used for phylogeny reconstruction. For comparison, the within-clade average protein sequence identity was 92.6%, 95.0% and 97.4% identity for NC64A, SAG and Pbi (excluding NE-JV-1) viruses, respectively. Between clades, the protein sequence identity ranged from 63.1% (NC64A vs. Pbi viruses) to 70.6% (Pbi vs. SAG viruses).

### Genome organization and gene colinearity

**Figure 2:**
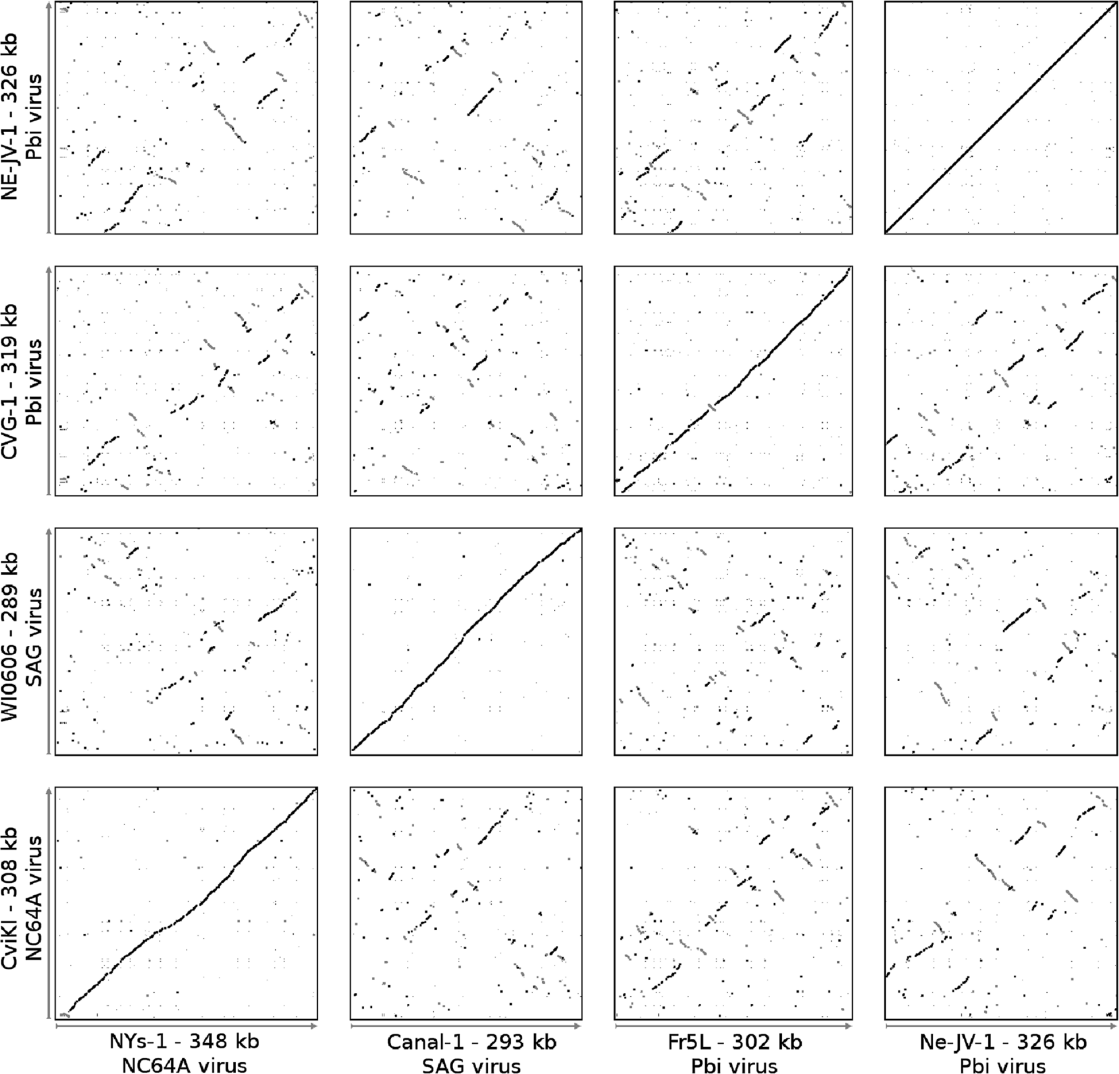
**Dot-plot alignments of ten newly sequenced *Chlorovirus *genomes.** Each dot represents a protein match between two viruses (BLASTP e-value < 1e-5) from genes in the same orientation (black) or in reverse orientation (gray). Best BLAST matches are shown with larger dots.

Figure 2 indicates that gene order is highly conserved among viruses infecting the same algal host, with only a few readily identifiable localized rearrangements, including inversions and indels (see below). Note that the order of contigs in assemblies was determined by maximizing sequence colinearity with the reference genomes. Indeed, 16 gaps were sequenced among six of the new viruses, the primers of which were designed based on the co-linearity of the previously sequenced chloroviruses; however, we cannot eliminate the possibility that additional inversion events exist if their boundaries precisely coincide with the contig ends. The high conservation of gene order contrasts strongly with the low residual gene colinearity between genomes from viruses infecting different algal hosts. The largest conserved genomic regions between CVs infecting different hosts encompassed 32 colinear genes. This observation is consistent with the reported high level of gene colinearity between the genomes of PBCV-1 and NY-2A, two NC64A viruses, as well as between those of MT325 and FR483, two Pbi viruses, but not between NC64A viruses and Pbi viruses [15,17]. We only found one exception to this rule: although the NE-JV-1 virus infects Pbi cells, its gene order is different from that of other Pbi infecting viruses. This lack of gene colinearity is consistent with the basal phylogenetic position of NE-JV-1 within the Pbi virus clade (Figure 1A). NE-JV-1 also has no long-range conserved gene colinearity with NC64A viruses or SAG viruses. This overall lack of colinearity with reference genomes was an issue when ordering the NE-JV-1 contigs between each other using the maximal sequence colinearity criterion. Thus, the order of contigs in the presented NE-JV-1 assembly must be taken with caution. In contrast, although the NC64A viruses also form two separate phylogenetic sub-groups – one sub-group contains PBCV-1 and the other NY-2A – genomes from both sub-groups share an almost perfect gene colinearity as exemplified by the dot-plot comparison between CviKI (PBCV-1 sub-group) and NYs-1 (NY-2A sub-group).

Gene order in *Mimiviridae* genomes is conserved toward the center of the genomes while significant disruptions of gene colinearity occur at the chromosome extremities [30]. This same conservation pattern occurs in *Poxviridae* genomes [31] suggesting that these two families of large DNA viruses, despite their considerable differences, might have evolved under common evolutionary processes linking replication and recombination. In contrast, no obvious differences were observed in the levels of conservation between the center and extremities of the CV genomes, suggesting a different mechanism of genome evolution in this viral clade. The levels of divergence between the colinear genomes of *Mimiviridae* and *Poxviridae* were comparable to the level of divergence between the most distant CV genomes that share no conserved gene colinearity; e.g., DNA polymerase proteins had 64% identical residues between *Mimivirus* and *Megavirus* (*Mimiviridae*) and 65% identical residues between deerpox and variola viruses (*Poxviridae*) [30], while the most divergent CV DNA polymerase protein pair shared 64% identical residues between the SAG virus OR0704.3 and NC64A virus MA-1D. Taken together, these observations suggest that at comparable genetic distances, genome rearrangements were more frequent in CVs than in *Mimiviridae* and *Poxviridae*.

Some spontaneous antigenic variants of PBCV-1 contained 27- to 37-kb deletions in the left end of the 330-kb genome [32]. Although these mutant viruses stably replicate in the *C. variabilis* host in laboratory conditions, albeit with phenotypic variations compared to the PBCV-1 wild type strain, it was unknown if such mutants existed in natural populations. The NC64A virus KS1B isolated in Kansas, USA contained a 35-kb deletion in the left end, when compared to the PBCV-1 wild type. This finding suggests that the deleted region that encompasses 29 ORFs in the PBCV-1 genome is dispensable in a natural environment. The missing PBCV-1 ORFs encode 2 capsid proteins, a pyrimidine dimer-specific glycosylase and 26 putative proteins with unknown function (Additional file 5: Table S3). Thus the KS1B virus may have altered capsid and DNA repair capability. Further study is required to determine if the KS1B genotype is common and stably fixed in the natural population or if it is a rare mutant that was sampled by chance or if it results from a recent mutation that occurred during maintenance of the virus in the laboratory.

### Origin of the CV genes

**Figure 3:**
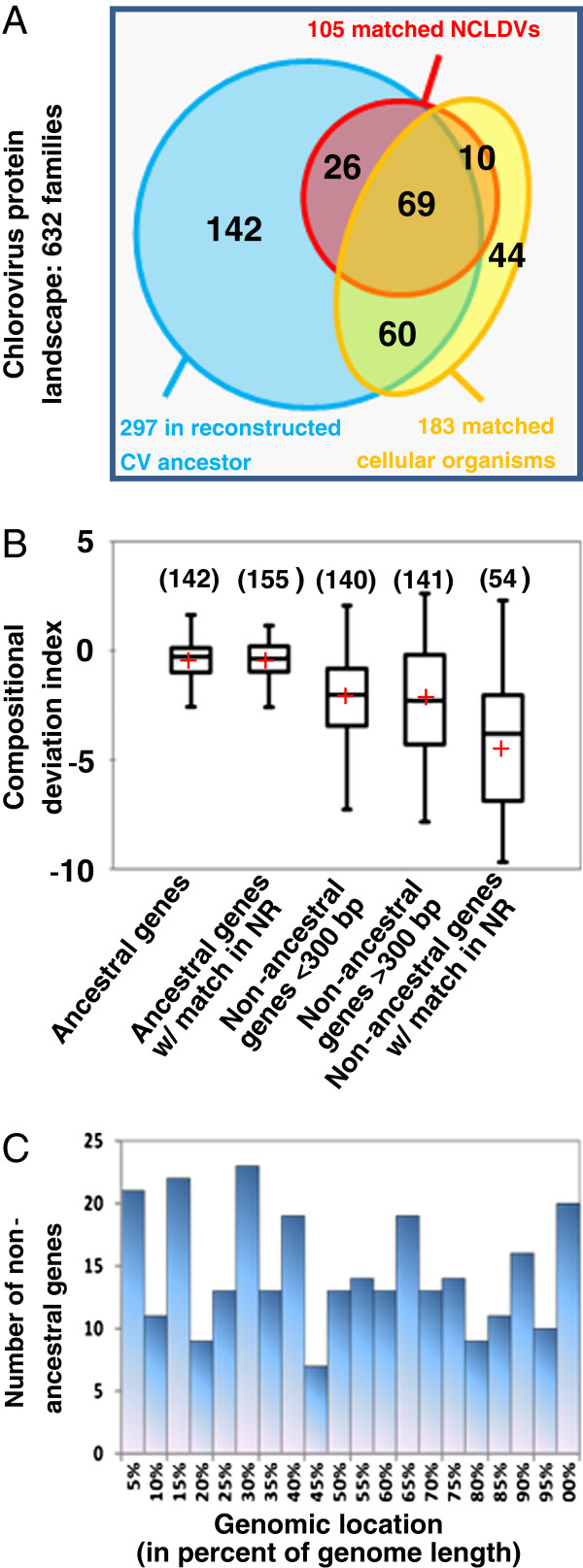
**Characteristics of *Chlorovirus *protein families. A**: Distribution of protein families in the ancestral and non-ancestral subsets. **B**: Box-plot distributions of median compositional deviation index (CDI) in gene families. The number of gene families in a category is given in parentheses. Distribution means are shown by a red cross; medians are shown by horizontal lines in boxes. **C**: Distribution of genomic locations of non-ancestral gene families. For each family, we recorded the average genomic location for gene members that occur in colinear genomes.

Reconstruction of ancestral genomes using the maximum parsimony method predicts that the last common ancestor of all sequenced CVs encoded at least 297 protein families (Figure 3A), including 155 core CV protein families plus 142 families that were lost in one or more modern CV genomes. This result suggests that the last common CV ancestor had a gene pool size slightly bigger than the extant viruses that encode 257 to 288 protein families (Table 1). The ancestral families account for 82% to 88% of the protein repertoire in the modern CVs. One hundred and five ancestral CV proteins also had homologs in other NCLDV genomes and were potentially inherited from an even older NCLDV ancestor; however, 335 (53%) of the 632 predicted chlorovirus protein families could not be traced back to the CV ancestor, which most probably also infected chlorella-like hosts. A fraction of them were presumably encoded in the ancestral genome and subsequently lost in all of the NC64A, Pbi and SAG viruses, so that their occurrence in the common ancestor could not be established using the parsimony criterion. Furthermore, we cannot rule out that some of the ORFan genes (ORF without match in sequence databases and the other chlorovirus sub-genera) are erroneous predictions. Sequence randomization between non-ORFan genes indicates that on average less than 1 ORF >300 bp in size can be obtained by chance in a chlorovirus genome; 185 non-ancestral protein families were encoded by ORFs that have a median length >300 bp. Alternatively, the corresponding genes could have been gained after the divergence of the main CV clades. There are three known mechanisms that can lead to gene gain: duplication of existing genes, capture of genes from other genomes through horizontal gene transfer (HGT) and creation of new genes from non-coding sequences *de novo*. Although gene duplicates exist in the CVs, they were not considered in subsequent analyses because in-paralogs were aggregated into existing orthologous clusters in the construction of the protein families.

### Non-ancestral genes

The oligonucleotide frequency in a sequence is known to be species-specific and can be used as a genomic signature [33]. Since DNA transfers originate from species with a compositional signature different from that of the recipient species, significant deviation of a signature between ORFs and the rest of the genome may signal recently transferred DNA. For each virus we constructed a five-order non-homogeneous Markov chain model of nucleotide frequency in the ORFs that were identified as being vertically inherited from the last common CV ancestor (i.e., ancestral ORFs). This model was used to compute a compositional deviation index (CDI) for ancestral and non-ancestral ORFs. The distributions of CDI values shown in Figure 3B differed significantly between ancestral and non-ancestral ORFs (Kruskal–Wallis test p < 0.0001 and Steel-Dwass-Critchlow-Fligner W* test p < 0.0001 between each pairwise combination of ancestral and non-ancestral CDI subsets). On average, non-ancestral ORFs had lower CDI values meaning that their nucleotide composition tends to exhibit a poorer fit to the nucleotide frequency model. This trend was true irrespective of the identification of homologs in databases or not. Furthermore, the distributions of CDI values for long (>300 bp) and short (<300 bp) ORFan families were not significantly different (Mann–Whitney test p ~0.99). This suggests that at least a fraction of the non-ancestral genes, including the genes with no recognizable homologs in the database, have been captured by HGT from genomes with distinct nucleotide compositional biases and that this event was sufficiently recent so that the difference in nucleotide composition is still visible.

To test this hypothesis, phylogenetic trees were reconstructed from 35 of the 54 non-ancestral protein families that had significant matches in Genbank. For the remaining 19 protein families, no reliable phylogenetic tree could be generated due to the scarcity of homologous sequences or too high sequence divergences between homologs. Most of the 35 phylogenetic trees were not conclusive as to the exact evolutionary history of the viral genes (Phylogenetic trees are shown in Additional file 6: Figure S3 and a summary of the interpretations is shown in Additional file 7: Table S4): In many cases, CV proteins had relatively deep branches in the tree implying that if the hypothesis of a recent HGT is supportable, sequences of the donor genome or its close relatives are not available in databases. Moreover, cellular homologs were sometimes sporadically distributed among eukaryotes, bacteria and sometimes viruses, and phylogenetic trees exhibited major discrepancies with the accepted phylogeny of the organism. Altogether these results suggest that these proteins are encoded by genes that were frequently exchanged between cellular organisms and between cellular organisms and viruses. In nine of the phylogenetic trees the CV proteins branched as a sister group to green algae or land plants. However, in only one case did the CV proteins directly branch on the *C. variabilis* branch, i.e., a tree topology consistent with a recent HGT between viruses and hosts. This HGT was readily identified as a capture of the algal dUDP-D-glucose 4,6-dehydratase gene by SAG viruses because the viral protein clade branched within the green algal phylogenetic sub-tree (CL0780 in Additional file 6: Figure S3). Thus, except for this obvious case, the origin of the green algal-like viral genes is unclear. Three alternative scenarios can explain this incongruence: (i) CVs captured green algal genes during infection of other algae that are distantly related to these hosts. However, this hypothesis is not consistent with the apparent specificity of CVs for one of the three algal strains. (ii) CVs captured genes from their “natural” algal host(s) but these genes have been lost in the genome of the model strain *C. variabilis* NC64A. (iii) CVs captured genes within the algal host from other parasites or symbionts (viruses or bacteria) that contain green algal genes. In fact, 18 phylogenetic trees placed CV proteins in a sister position to bacteria. For six of the concerned protein families, homologs were also found in phages or other DNA viruses.

Thus, although the non-ancestral genes exhibit specific compositional features suggesting this subset is enriched in sequences with a potential extraneous origin, a majority of them (281 families) have no identifiable homolog in the databases, and for those that do (54 families), only a few produced a phylogenetic tree where the clade of the donor organism could be identified with a reasonable degree of confidence. Thus, if the hypothesis of acquisition by HGT is supported for the non-ancestral CV genes, they must originate from a DNA fraction that is under-represented in public databases.

Finally, we investigated the location of the non-ancestral genes within the CV genomes. The non-ancestral genes are evenly distributed across the CV genomes (Figure 3C). This contrasts with the cases of *Mimiviridae* and *Poxviridae*, which have genus- and species-specific genes clustered toward the extremities of their genomes, whereas the conserved genes are clustered in the middle [30,34]. This result reinforces the apparent differences between the evolution of CV genomes and that of the members of other NCLDV clades.

### Gene exchanges with the algal host

Previous studies attempted to identify genes of cellular origin in CV genomes [35]. It was estimated that 4 to 7% of CV genes are of bacterial origin, and an additional 1 to 2% were acquired from the plant lineage [36] though interpretation of the results was subject to controversy [37]. These low numbers put into question the real significance of HGT in CVs; however, the genome of the host for the NC64A viruses was not sequenced at the time of these previous studies. Since the release of the *C. variabilis* genome sequence [38], no systematic study of gene exchanges between CVs and the algal host has been undertaken. It should be noted that the SAG virus host, *C. heliozoae*, and Pbi host, *M. conductrix*, have not been sequenced. However, their close phylogenetic relationships with the host for the NC64A viruses permit using the *C. variabilis* genome as a proxy for the other host species. The above analysis of the non-ancestral protein families already identified a case of gene acquisition by SAG viruses from the host; we completed this study by investigating the phylogenetic affinities in the ancestral protein family subset.

Out of the 297 ancestral families, 42 had significant matches with *C. variabilis* homologs. Subsequent phylogenetic analysis identified seven families where the viral protein clades branched next to *C. variabilis* homologs, reflecting potential HGT between viruses and the host (Additional file 8: Figure S4). For two of them, the likely direction of HGT could be inferred as a capture of the algal gene by the CV ancestor based on the placement of the CV branch within the green algae clade. These 2 genes encode a translation elongation factor EF-3 (CL0450) and an unknown protein (CL0511). In yeast, EF-3 interacts with both ribosomal subunits and facilitates elongation factor EF-1-mediated cognate aminoacyl-tRNA binding to the ribosomal A-site [39]. Thus, capture of the algal EF-3 gene may help CVs by enhancing protein biosynthesis during infection. For the 4 remaining families (chitin deacetylase, chitinase and 2 unknown proteins), *C. variabilis* is the only plant organism to share these viral genes; thus their vertical inheritance from an ancestor is more unlikely as this would imply many subsequent gene losses among the other descendants of the plant ancestor. An alternative scenario involves gene captures by the algal host from the virus genome. Although no lysogenic cycle has yet been identified among CVs, some members of the phycodnavirus family are known to integrate into the host genome [40]. Thus, these algal genes may correspond to remnants of ancient integrated genomes of unknown lysogenic viruses.

Altogether, these results suggest that the CVs and their hosts did not frequently exchange genes. Overall, only 3 genes showed evidence of capture through host-to-virus exchanges and in 4 other genes the opposite scenario is more likely (virus-to-host exchange). Furthermore, 2 of the host-to-virus exchanges occurred before the divergence of the CVs (i.e., in ancestral protein families), suggesting that they could have contributed to the early adaptation of viruses to their algal host. Thus, although large viruses are often presented as mainly evolving by recruiting genes from their hosts, this conjecture does not hold true for the CVs.

## Conclusion

One of the most striking findings from this study is that more than half of the CV predicted protein families are encoded by genes of recent extrinsic origin (after Chlorovirus divergence) – most of which are also ORFans. The proportion of non-ancestral genes in individual CV genomes is substantial–12% to 18% of the protein families–though this proportion is similar to atypical genes of likely extrinsic origin in bacterial genomes [38]; however clues as to the potential donor genomes are lacking. The algal host cytoplasm is probably the sole milieu where the viral genome is accessible for recombination and acquisition of extrinsic genes. Consequently horizontally transferred genes can arise from 3 potential sources: (i) host DNA, (ii) bacterial DNA, and (iii) DNA from other (perhaps distantly related) viruses competing for the same algal host.

Our study shows that the capture (and fixation) of algal host DNA has been rare in the evolutionary history of CVs and cannot explain the vast majority of non-ancestral CV genes. Furthermore, we believe that bacterial DNA is not a major source of extrinsic genes in CVs: if non-ancestral genes were mainly of bacterial origin we would expect that the proportion of ORFans in the non-ancestral gene data set to be comparable to the proportion of ORFans in bacterial genomes. Estimated frequencies for ORFans in bacterial genomes vary between 7% for the most recent estimates [41] to 20–30% for estimates made early in the history of genome analysis [42], when only the first organisms had been sequenced. These frequencies are significantly below the frequency of ORFans in the non-ancestral CV protein family dataset (from 141/195 = 72% if we only consider “long” ORFans to 281/335 = 84% if we consider all predicted genes).

Thus if the conjecture of acquisition by HGT is true for the non-ancestral CV genes, they must originate from a still largely un-sequenced reservoir of genomes. The biological entities that match best with this characteristic are the viruses themselves. Viruses are by far the most abundant entities in aquatic environments and we are only now realizing the extraordinary range of global viral biodiversity [8]. Thus, we suspect that the apparent incongruence between compositional evidence of HGT and lack of donor (or close relative) sequences in the databases reflect the fact that non-ancestral CV genes arose from recombination with other unknown viruses that infect the same hosts. However, this does not rule out alternate hosts that could be underrepresented in the existing databases as possible donors.

## Methods

### Virus isolation and storage

The set of viruses used in this study were collected at different times over several years from various terrestrial waters around the world (see Additional file 9: Table S5). The water samples were evaluated for plaque-forming viruses on the specific algal host, and the plaque isolates were chosen based on phenotypic characteristics of interest or for geographic distribution purposes. The intention was to evaluate a broad spectrum of chloroviruses with approximately an equal number infecting each of the three algal hosts. The plaque isolates were plaque purified at least two times, then amplified in liquid culture for the purposes of virus purification using the method previously described [14]. The purified viruses were plaque assayed to determine the number of infectious particles and stored at 4°C.

### DNA isolation

Viral DNA was purified from virions that had been treated with DNase I (10 units/ml in 50 mM Tris–HCl pH 7.8/1 mM CaCl_2_/10 mM MnCl_2_ at 37°C for 1 hr), using the UltraClean®Blood DNA Isolation Kit (MO BIO Laboratories, Carlsbad, CA). The DNA was evaluated for quantity and quality by measuring absorbance at 260 and 280 nm with a Thermo Scientific NanoDrop 2000 spectrophotometer, and by measuring fluorescence of dye-augmented DNA using the PicoGreen and a Qubit fluorometer (Invitrogen, Carlsbad, California).

### Genomic library preparation and sequencing

Genomic libraries were constructed from pairs or triplets of pooled viral genomic DNA. A schematic representation of the multiplexed sequencing pipeline is shown in Additional file 10: Figure S5. Using the Roche Rapid Library Preparation method for GS FLX Titanium chemistry (Roche 454 Life Sciences, Branford, Connecticut), sample DNA was fragmented by nebulization. DNA fragments were end repaired with polynucleotide kinase and T4 DNA polymerase, then purified by size exclusion chromatography. Selected DNA fragments were ligated to a Rapid Library Multiplex Identifier (MID) adaptor designed for GS FLX Titanium chemistry. The MID adaptors were designed with a unique decamer sequence to facilitate multiplex sequencing with the 454 technology, such that the resulting library reads can be reliably sorted after sequencing using SFF software tools. MID adaptor ligated DNA fragments were again size selected by chromatography, quantified with a TBS-380 mini-fluorometer (Promega, Madison, Wisconsin). The Rapid Library quality was assessed with an Agilent Bioanalyzer High Sensitivity DNA chip (Agilent Technologies, Santa Clara, California). The average fragment length was between 600 bp and 900 bp, with the lower size cut-off at less than 10% below 350 bp. Pooled DNAs were titrated to obtain the optimal copies per bead (cpb). After titration, 3 cpb was chosen as the best DNA and bead ratio and corresponding amounts of DNA were added to the subsequent emPCR reactions. EmPCR was performed with the 454/Roche Lib-L (LV) kits following manufacturer's protocol for the Roche 454 GS FLX Titanium.

### Sequence assembly and gene prediction and annotation

All of the viral DNA genomic libraries, as emPCR products, were sequenced through two duplicated multiplex runs on a Roche GS FLX Titanium sequencer. 454 image and signal processing software v.2.3 generated a total of 2,434,736 PassedFilter reads after removing reads under 40 bp in length. The raw data from the 454-pyrosequencing machine were first processed through a quality filter and only saved sequences that met the following criteria: i) contained a complete forward primer and barcode, ii) contained no more than one “N” in a sequence read where N is equivalent to an interrupted and resumed signal from sequential flows, iii) reads were 200 to 500 nts in length, and iv) reads had a average quality score of 20. Using SFF tools implemented in the 454 GS-Assembler 2.3, each read was trimmed to remove 3’ adapter and primer sequences and was parsed by a MID adaptor barcode. The corresponding QUAL file also was updated to remove quality scores from reads not passing quality filters. This procedure allowed the unambiguous assignment of 2,429,860 reads of 384-bp on average to the corresponding genomic libraries

Separate assembly for each library was performed by the MIRA assembler version 3.2.0 using the following parameters: --job = denovo, genome, accurate, 454 -DP:ure = yes -CL:emrc = yes -AL:mo = 50 -ED:ace = yes. Overall a total of 1557 contigs containing 2,330,493 reads were generated.

The resulting contigs were assigned to their corresponding viruses and ordered between each other by alignment against reference viral genomes, e.g. PBCV-1, NY-2A, and AR158 for NC64A viruses [GenBank:JF411744, DQ491002, DQ491003], ATCV-1 for SAG viruses [GenBank:EF101928] and MT325 and FR483 for Pbi viruses [GenBank:DQ491001, DQ890022].

A first list of putative ORFs was constructed using the GeneMarkS program (using the -lo and -op options) [43]. A list of potential ORFs (size >60 codons) occurring in the intergenic regions between GeneMarkS predicted genes was compiled. These potential ORFs were added to the predicted gene list only if they had a significant match (BLASTP e-value < 1e-5) in the Genbank non-redundant (nr) database, omitting matches in the *Chlorovirus* genus. Predicted proteins were functionally annotated based on match against multiple sequence databases, including Swissprot, COG, Pfam and nr databases using an e-value threshold of 1e-5 for both BLASTp and HMMer searches. tRNAs genes were predicted using the tRNAscan-SE program, ignoring pseudo- and undetermined-tRNAs.

### Protein clustering

Putative orthologous protein pairs were first identified using the reciprocal best BLASTp hit criterion and assembled into orthologous clusters by the single-linkage clustering method. Putative orthologous proteins of four sequenced *Ostreococcus* viruses were also included in the clustering scheme to serve as an outgroup in subsequent analyses. In-paralogs (resulting from the duplication of a protein gene after divergence of two viral lineages) were assigned to existing orthologous clusters if their alignment scores with one protein of a cluster were greater than any of the alignment scores between this protein and the other members of the cluster.

### Phylogenetic analysis

Phylogenetic analysis was performed using the following general pipeline: homologous sequences were searched in databases using the BLAST EXPLORER tool [43]. Multiple-sequence alignments were performed using the MUSCLE program [44], followed by manual edition, and removal of gaped sites and poorly aligned regions. Phylogenetic trees were reconstructed using the PHYML program (Maximum likelihood) [45] and Mega 4 (Neighbor Joining and maximum parsimony) [46]. Statistical support for branches was assessed using 1000 bootstrap datasets.

### Chlorovirus ancestor gene content

Given the phylogeny of the sequenced CV shown in Figure 1A, protein families that contained at least one member in one of the NC64A viruses and at least one member in one of the Pbi viruses or SAG viruses were considered as being inherited from the last common CV ancestor. A total of 290 protein families were identified as “ancestral” by this procedure. In addition, 7 protein families that are a sister group to homologs in NCLDV in phylogenetic ML trees were considered to be inherited from the last common ancestor. Thus the genome of the last common CV ancestor was inferred to encode at least 297 protein families.

### Compositional deviation index

To distinguish between intrinsic and extrinsic genes, a compositional deviation index (CDI) was computed. The CDI score reflects how much the nucleotide composition of an ORF deviates from that of a reference set of ancestral ORFs. Thus, an extrinsic ORF integrated into the genome is distinguished from the recipient genome sequences by the nucleotide composition, unless the donor and recipient species are close relatives with similar nucleotide compositional biases. Ancient transferred genes may be indistinguishable, because the nucleotide composition of horizontally transferred genes generally converges with that of the recipient genome by mutation pressure. Thus, this procedure preferentially detects recent horizontally transferred genes for which the compositional convergence process has not been completed.

Our method for computing CDI scores was largely inspired from earlier works on gene finding [47] and extrinsic DNA identification [48]; these two references contain detailed explanations of the statistical framework and construction of the model. A non-homogenous Markov model for ancestral coding nucleotide sequences was defined by four components: P^0^, the initial probability vector for starting k-bp tuples j in ancestral ORFs, and P^1^, P^2^, P^3^, three transition matrices that define the probability that a k-tuple j whose first nucleotide occupies respectively the f = 1^st^, 2^d^ or 3^th^ position in a codon, is followed by one of the four possible nucleotides (i). The likelihood of finding an ORF of length l given the model is:

$$P\left({\mathrm{ORF}|\mathrm{COD}}_{\mathrm{anc}}\right)=P^{0}\left(j_{1}\right)P^{1}\left(i_{k+1}|j_{1}\right)P^{2}\left(i_{k+2}|j_{2}\right)P^{3}\left(i_{k+3}|j_{3}\right)\ldots P^{f}\left(i_{l}|j_{l-k}\right)$$

Numerical values of the parameters of the model (P^0^, P^1^, P^2^ and P^3^) were derived from the count of k-tuples N_j_ and (k + 1)-tuples N_(j,i)_ in the training sequence set containing all ancestral ORF of a CV. That is, initiation probabilities were taken as the frequencies of k-bp tuples, and transition probabilities were equal to $N_{\left(j,i\right)}^{f}/N_{\left(j\right)}^{f}\text{.}$ The order of the Markov chains was set to five (k = 5) to avoid an overfitting of the parameters.

For each ORF, the CDI value was computed as follows: first the mean and standard deviation (SD) of P(ORF_r_|COD_anc_) for 100 random coding sequences emitted from the Markov chain model was determined. The random sequences had the same length that the ORF for which CDI was computed. The CDI was calculated according to the formula:

$${\mathrm{CDI}}_{\mathrm{ORF}}=\frac{P\left({\mathrm{ORF}|\mathrm{COD}}_{\mathrm{anc}}\right)-\bar{P}\left({\mathrm{ORF}}_{r}|{\mathrm{COD}}_{\mathrm{anc}}\right)}{{\mathrm{SD}}_{P\left({\mathrm{ORF}}_{r}|{\mathrm{COD}}_{\mathrm{anc}}\right)}}$$

The expectation is CDI = 0 for ORFs with nucleotide compositions that fit with the model for ancestral coding nucleotide sequences, while ORFs whose nucleotide composition significantly deviates from the model shall have CDI ≠ 0.

## Competing interests

The authors declare that they have no competing interests.

## Authors’ contributions

AJ: analyzed the data and drafted the manuscript. DDD: Conceived the project, provided several viruses, purified the viruses and the viral DNAs, developed the sequencing libraries, contributed to the sequence assembly and contig assignment, contributed to the data analysis and interpretation and writing of the manuscript. JRG, IA, MK, JV, ML provided essential materials for virus isolation and production. GD, OWM provided essential computational support for virus sequence analyses. JMC: helped to draft the manuscript. JLVE: initiated the project and helped write the final paper. GB: coordinated analysis of the data and drafted the manuscript. All authors read and approved the final manuscript.

## Supplementary Material

Additional file 1: Table S1. 632 *Chlorovirus* protein families. (XLSX 118 kb) Click here for file

Additional file 2: Table S2. Example of orthologous protein clusters viewed for the first time in Chloroviruses. (PDF 16 kb) Click here for file

Additional file 3: Figure S1. Neighbor joining tree of the reference concatenated alignment. The NJ tree of chloroviruses is based on a concatenated alignment of 32 core protein families (7762 gap-free sites). Phylogenetic distances were computed using the WAG + G + I substitution model. Branch support was estimated from 1000 bootstrap replicates. We only show bootstrap values < 90%. Branches that differed from the ML and MP trees are colored in red. (PDF 16 kb) Click here for file

Additional file 4: Figure S2. Maximum parsimony tree of the reference concatenated alignment. The MP tree of chloroviruses is based on a concatenated alignment of 32 core protein families (7762 gap-free sites). Phylogenetic tree was computed using the close-neighbor-interchange method. Branch support was estimated from 1000 bootstrap replicates. We only show bootstrap values <90%. (PDF 14 kb) Click here for file

Additional file 5: Table S3. PBCV-1 genes missing in the KS1B genome as the result of a 35Kb deletion. (PDF 20 kb) Click here for file

Additional file 7: Table S4. Sister groups to non-ancestral *Chlorovirus* proteins based on 35 phylogenetic trees shown in Additional file 7: Figure S3. Click here for file

Additional file 6: Figure S3. 35 phylogenetic trees of non-ancestral *Chlorovirus* protein families. Trees were reconstructed using the ML method using the WAG + G + I substitution model. Interior branch support was estimated by the approximate likelihood ratio test (aLRT). For the sake of clarity, we only show branch support for important clades. Taxon names are colorized according to taxonomic information: *Chlorovirus* (red), chlorophytes (dark green), streptophytes (light green), eukaryote (violet), prokaryote (pink) and DNA virus (blue). Genbank gi numbers are given after species names. Protein family ID and functional annotation are given above each tree. ) Click here for file

Additional file 8: Figure S4. Phylogenetic trees showing potential HGT between chloroviruses and *Chlorella*. Trees were reconstructed using the ML method using the WAG + G + I substitution model. Interior branch support was estimated by the approximate likelihood ratio test (aLRT). For the sake of clarity, we only show branch support for important clades. Taxon names are colorized according to taxonomic information: *Chlorovirus* (red), chlorophytes (dark green), streptophytes (light green), eukaryote (violet), prokaryote (pink) and DNA virus (blue). Genbank gi numbers are given after species names. Protein family ID and functional annotation are given above each tree. Click here for file

Additional file 9: Table S5. Attributes of the sequenced chloroviruses. Click here for file

Additional file 10: Figure S5. Schema of the multiplexed sequencing strategy. Click here for file
