# Supplementary material for: Towards defining the chloroviruses: a genomic journey through a genus of large DNA viruses
Source: BMC Genomics. 2013 Mar 8;14:158. doi: 10.1186/1471-2164-14-158 (PMC3602175; doi:10.1186/1471-2164-14-158)
Supplement: Additional file 2: Table S2. — Example of orthologous protein clusters viewed for the first time in Chloroviruses. (PDF 16 kb) [file 1471-2164-14-158-S2.pdf]

Table S2: Examples of orthologous protein clusters viewed for the first time in Chloroviruses.

Cluster ID	Putative function
CL0049	Fumarate reductase
CL0462	Acetyltransferase
CL0875	SAM-dependent Methyltransferase
CL0940	Nitroreductase
CL0963	Glycosyl hydrolase
CL1018	Helicase
