# Supplementary figures and images for: Towards defining the chloroviruses: a genomic journey through a genus of large DNA viruses
Source: BMC Genomics. 2013 Mar 8;14:158. doi: 10.1186/1471-2164-14-158 (PMC3602175; doi:10.1186/1471-2164-14-158)

Figure S1

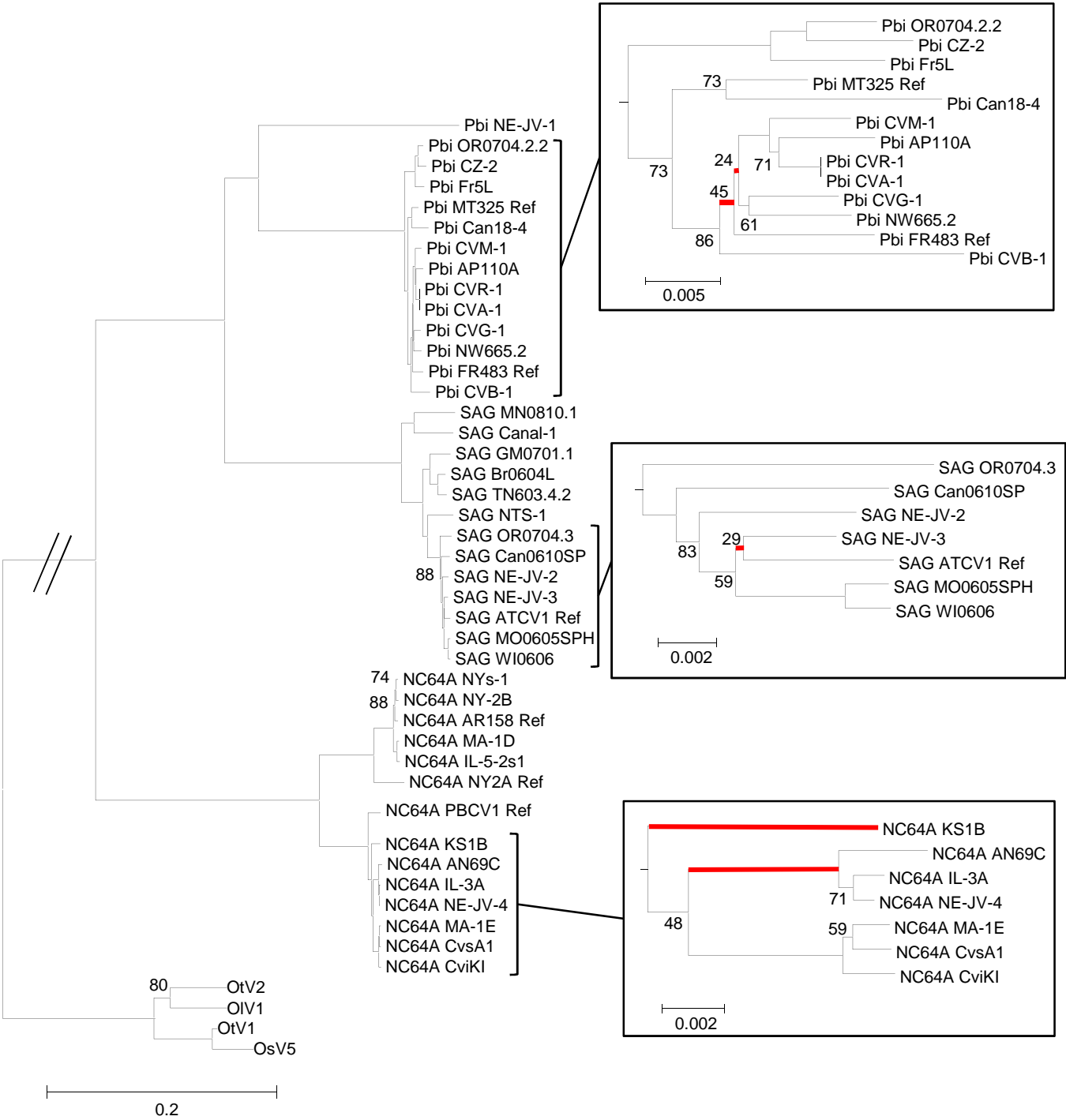

Supplement: Additional file 3: Figure S1. — Neighbor joining tree of the reference concatenated alignment. The NJ tree of chloroviruses is based on a concatenated alignment of 32 core protein families (7762 gap-free sites). Phylogenetic distances were computed using the WAG + G + I substitution model. Branch support was estimated from 1000 bootstrap replicates. We only show bootstrap values < 90%. Branches that differed from the ML and MP trees are colored in red. (PDF 16 kb) [file 1471-2164-14-158-S3.pdf]

Figure S2

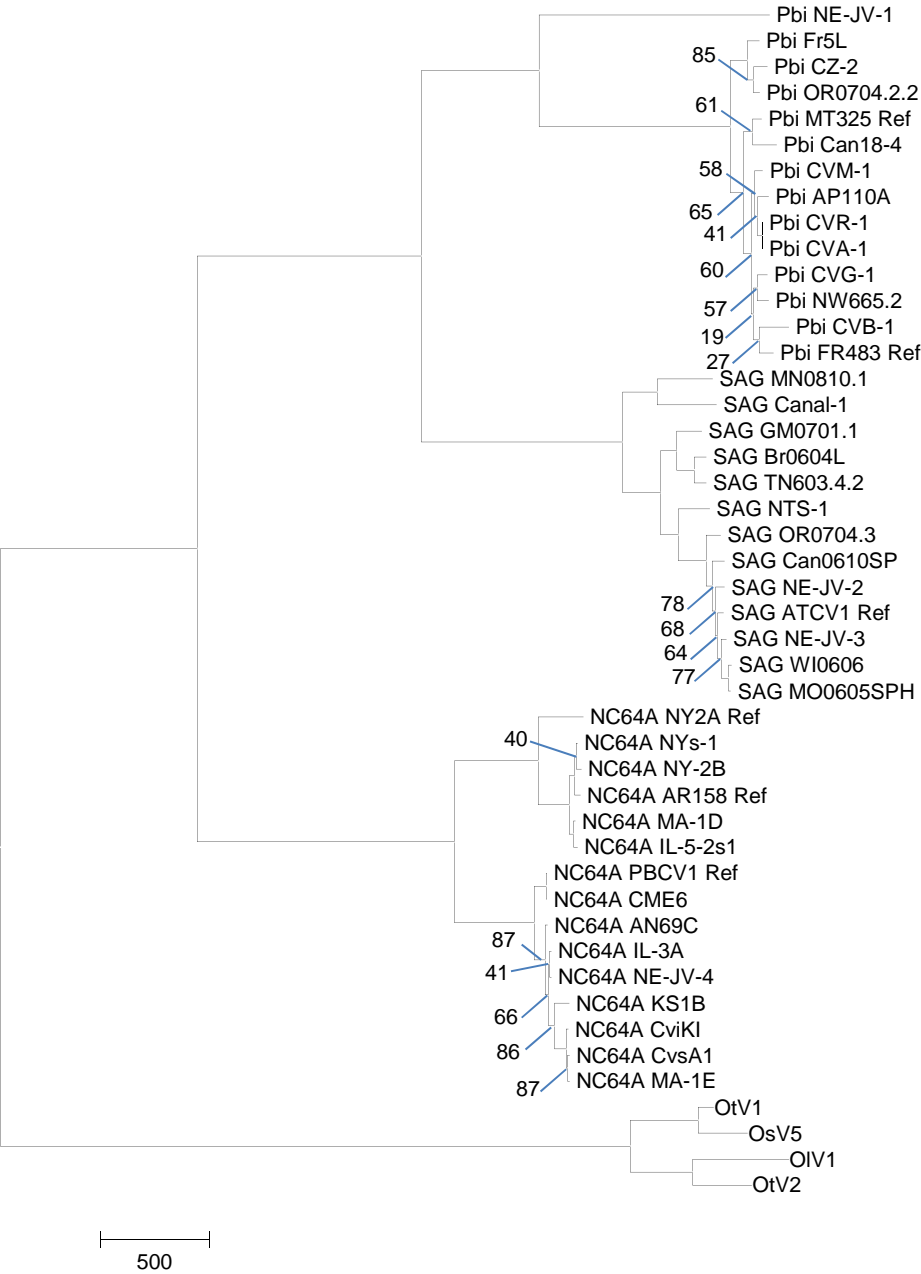

Supplement: Additional file 4: Figure S2. — Maximum parsimony tree of the reference concatenated alignment. The MP tree of chloroviruses is based on a concatenated alignment of 32 core protein families (7762 gap-free sites). Phylogenetic tree was computed using the close-neighbor-interchange method. Branch support was estimated from 1000 bootstrap replicates. We only show bootstrap values <90%. (PDF 14 kb) [file 1471-2164-14-158-S4.pdf]

Figure S5

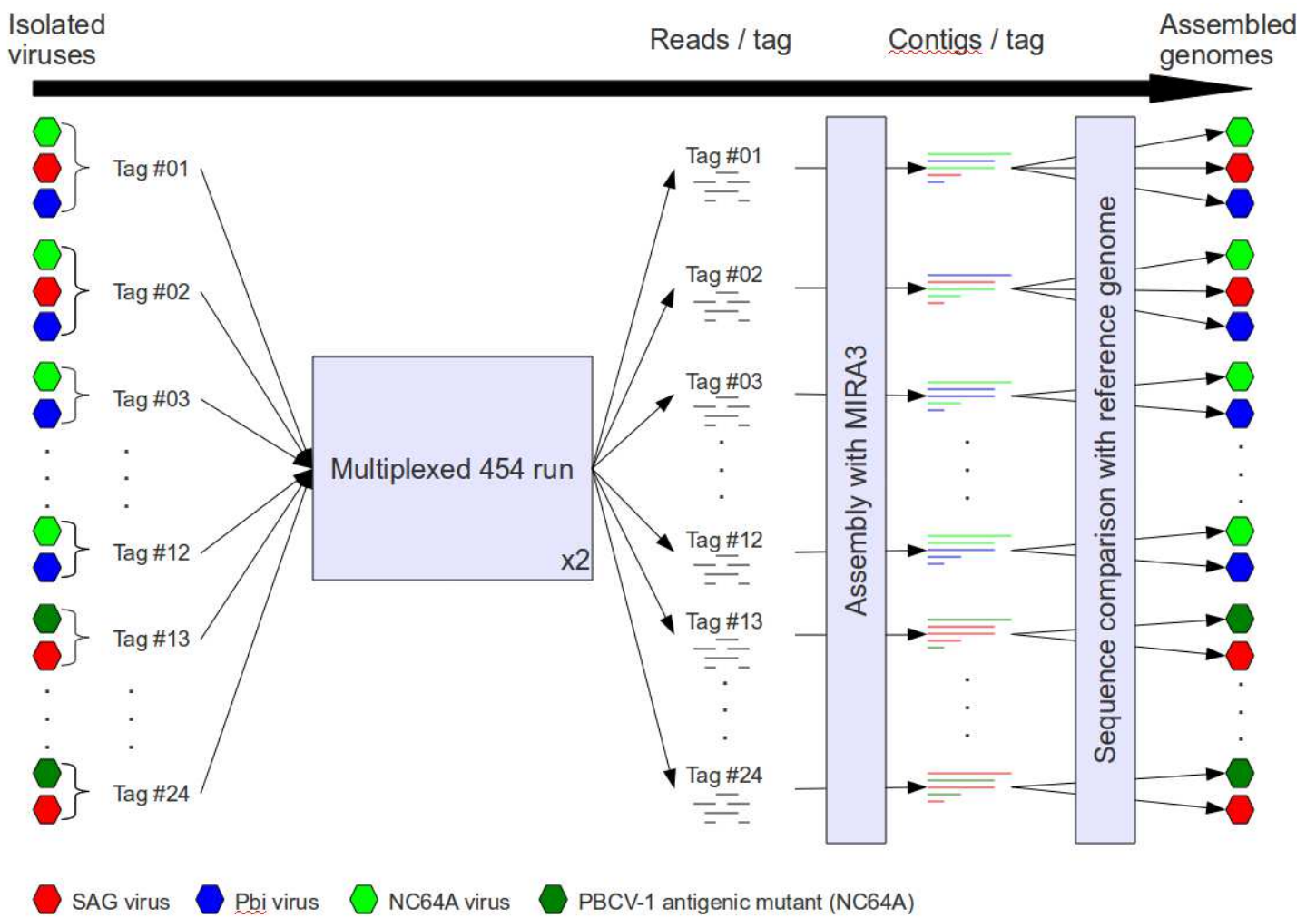

Supplement: Additional file 10: Figure S5. — Schema of the multiplexed sequencing strategy. [file 1471-2164-14-158-S10.pdf]
