# Supplementary material for: Towards defining the chloroviruses: a genomic journey through a genus of large DNA viruses
Source: BMC Genomics. 2013 Mar 8;14:158. doi: 10.1186/1471-2164-14-158 (PMC3602175; doi:10.1186/1471-2164-14-158)
Supplement: Additional file 5: Table S3. — PBCV-1 genes missing in the KS1B genome as the result of a 35Kb deletion. (PDF 20 kb) [file 1471-2164-14-158-S5.pdf]

Table S3 : PBCV-1 genes missing in the KS1B genome as the result of a 35Kb deletion

PBCV-1 gene name	Predicted function
A002L	Unknown protein
A002bL	Hypothetical protein
A002cR	Hypothetical protein
A003R	Unknown protein
A005R	Unknown protein
A007/008L	Unknown protein
A009R	Unknown protein
A010R	Capsid protein
A011L	Capsid protein
A014R	Unknown protein
A018L	Unknown protein
A025/027/029L	Unknown protein
A034R	Protein kinase
A035L	Unknown protein
A037L	Unknown protein
A039L	Unknown protein
A041R	Unknown protein
A044L	Unknown protein
A048R	Unknown protein
A049L	Unknown protein
A050L	Pyrimidine dimer-specific glycosylase
A050aL	Hypothetical protein
A051L	Unknown protein
A053R	Unknown protein
A058L	Hypothetical protein
A057aR	Hypothetical protein
A060L	Unknown protein
A061L	Unknown protein
A063L	Unknown protein
