# Supplementary material for: Towards defining the chloroviruses: a genomic journey through a genus of large DNA viruses
Source: BMC Genomics. 2013 Mar 8;14:158. doi: 10.1186/1471-2164-14-158 (PMC3602175; doi:10.1186/1471-2164-14-158)
Supplement: Additional file 7: Table S4. — Sister groups to non-ancestral Chlorovirus proteins based on 35 phylogenetic trees shown in Additional file 7: Figure S3. [file 1471-2164-14-158-S7.pdf]

Table S4: sister groups to non-ancestral CV proteins based on 35 phylogenetic trees shown in Figure S3

Cluster ID	Sister group	Function
CL0007	**Tetrahymena thermophila (Alveolata)**	ADP-ribosyl glycohydrolase
CL0011	**Phytophthora sojae (stramenopiles)**	unknown
CL0037	**Bacteria**	beta-lactamase
CL0049	**Chlorophyta**	NADH-dependent fumarate reductase
CL0055	**Bacteria**	unknown
CL0056	Mixed eukaryotes and bacteria	unknown
CL0063	**Plantae**	unknown
CL0065	**Bacteria**	unknown
CL0222	**Bacteria**	DNA methylase
CL0356	**Oceanicola granulosus (Bacteria)**	methyltransferase
CL0375	**Coccomyxa subellipsoidea (Chlorophyta)**	glycosyltransferase
CL0466	**Chlorophyta**	MIP family channel protein
CL0482	**Bacteria**	glutaredoxin
CL0489	**Actinobacterium (Bacteria)**	unknown
CL0503	**Bacteria**	unknown
CL0533	Basal	unknown
CL0561	**Polynucleobacter necessarius (Bacteria)**	Glycosyltransferase family 17
CL0607	**Waddlia chondrophila (Bacteria)**	methyltransferase
CL0624	**Chlorophyta**	THYLAKOID FORMATION 1; inositol phosphatase-like protein
CL0739	Basal	aspartate carbamoyltransferase
CL0767	**Aureococcus anophagefferens (diatom)**	unknown
CL0778	**Viridiplantae**	unknown
CL0780	**Chlorella (Chlorophyta)**	dUDP-D-glucose 4,6-dehydratase
CL0787	**Viridiplantae**	Potassium transporter
CL0792	**Kytococcus sedentarius (Bacteria)**	mannose-6-phosphate isomerase
CL0796	**Chlorophyta**	ribonucleoside-triphosphate reductase
CL0875	Basal	methyltransferase
CL0876	**Brevundimonas diminuta (Bacteria)**	methyltransferase
CL0878	**Prochlorococcus marinus (Bacteria)**	dTDP-glucose pyrophosphorylase/HAD superfamily hydrolase
CL0879	**Prochlorococcus marinus (Bacteria)**	unknown
CL0940	**Actinobacterium (Bacteria)**	Nitroreductase
CL0957	**Brevibacillus brevis (Bacteria)**	NADH-dependent oxidoreductase
CL0963	Mixed eukaryotes and bacteria	glycosyl hydrolase
CL0978	**Streptophyta**	unknown
CL0989	**Bacteria**	unknown
