# Supplementary material for: Towards defining the chloroviruses: a genomic journey through a genus of large DNA viruses
Source: BMC Genomics. 2013 Mar 8;14:158. doi: 10.1186/1471-2164-14-158 (PMC3602175; doi:10.1186/1471-2164-14-158)
Supplement: Additional file 6: Figure S3. — 35 phylogenetic trees of non-ancestral Chlorovirus protein families. Trees were reconstructed using the ML method using the WAG + G + I substitution model. Interior branch support was estimated by the approximate likelihood ratio test (aLRT). For the sake of clarity, we only show branch support for important clades. Taxon names are colorized according to taxonomic information: Chlorovirus (red), chlorophytes (dark green), streptophytes (light green), eukaryote (violet), prokaryote (pink) and DNA virus (blue). Genbank gi numbers are given after species names. Protein family ID and functional annotation are given above each tree. ) [file 1471-2164-14-158-S6.pdf]

Figure S3

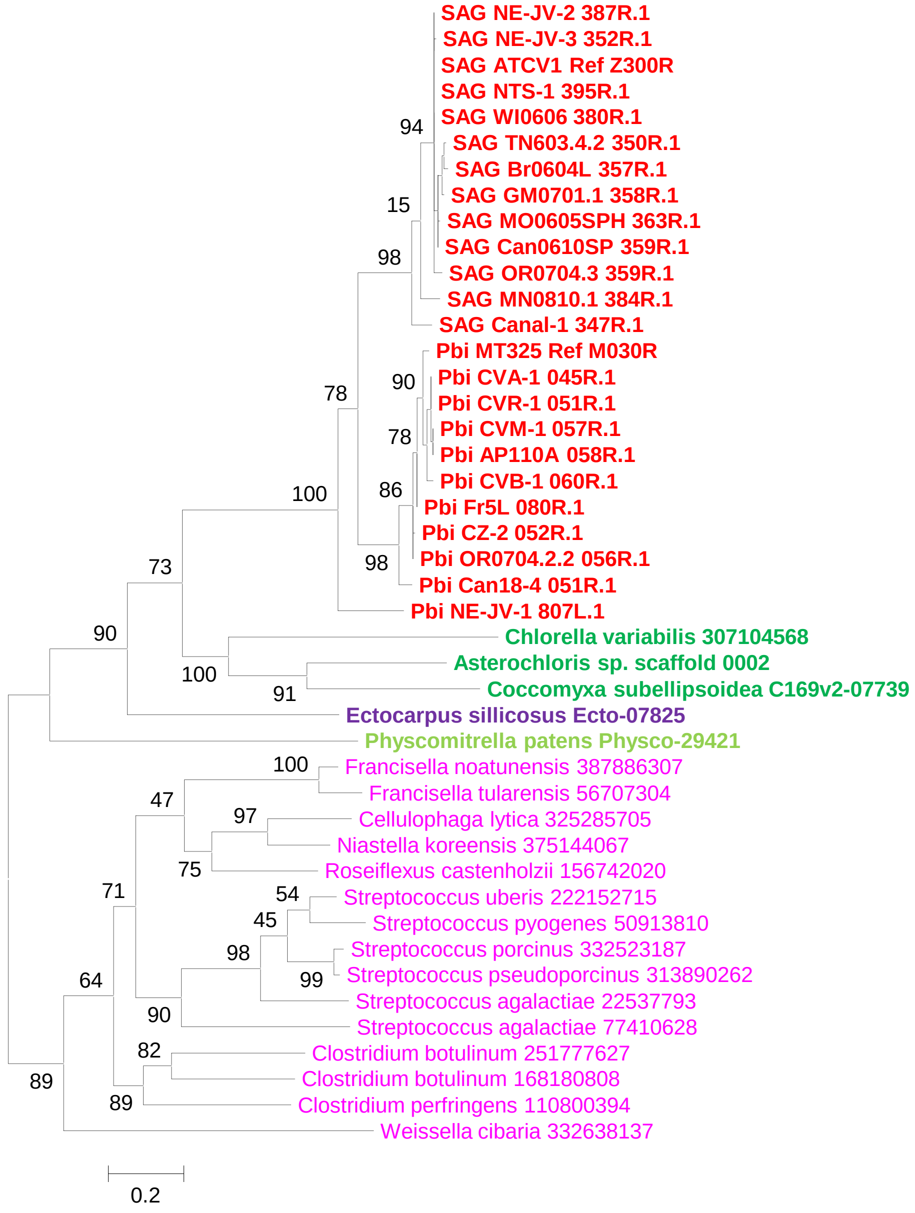

CL0466: MIP family channel protein

CL0780: dUDP-D-glucose 4,6-dehydratase

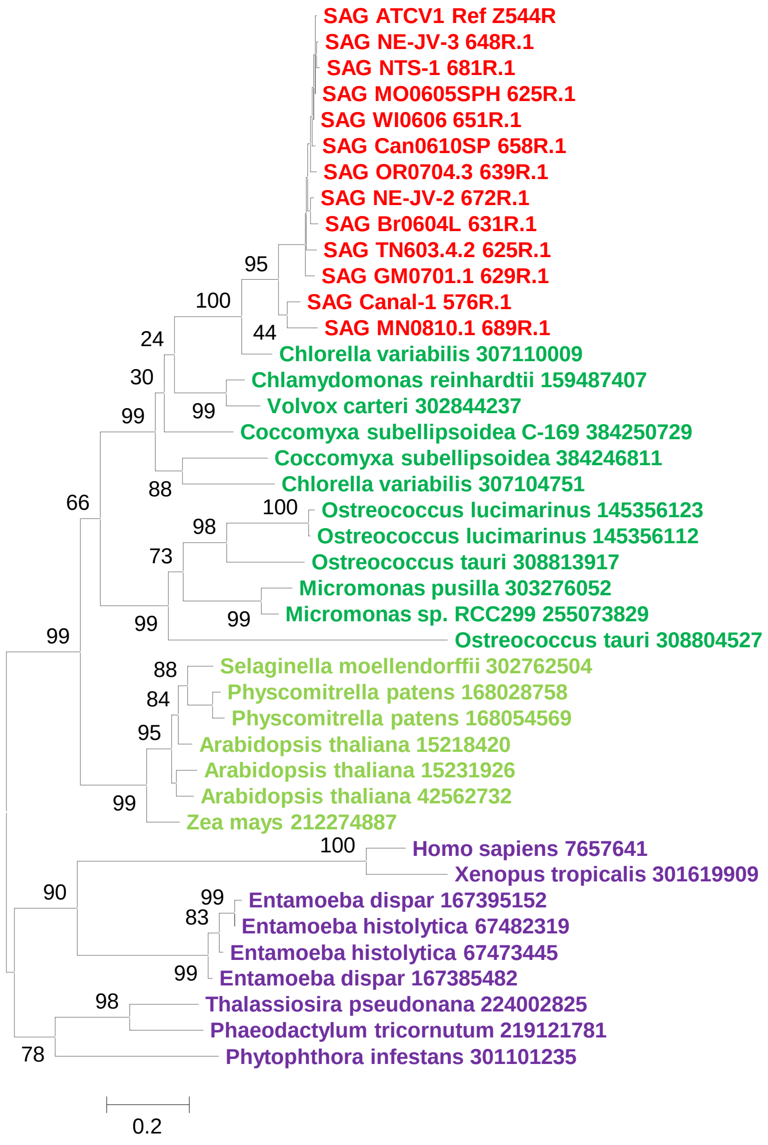

Figure S3: continued

CL0049: NADH-dependent fumarate reductase

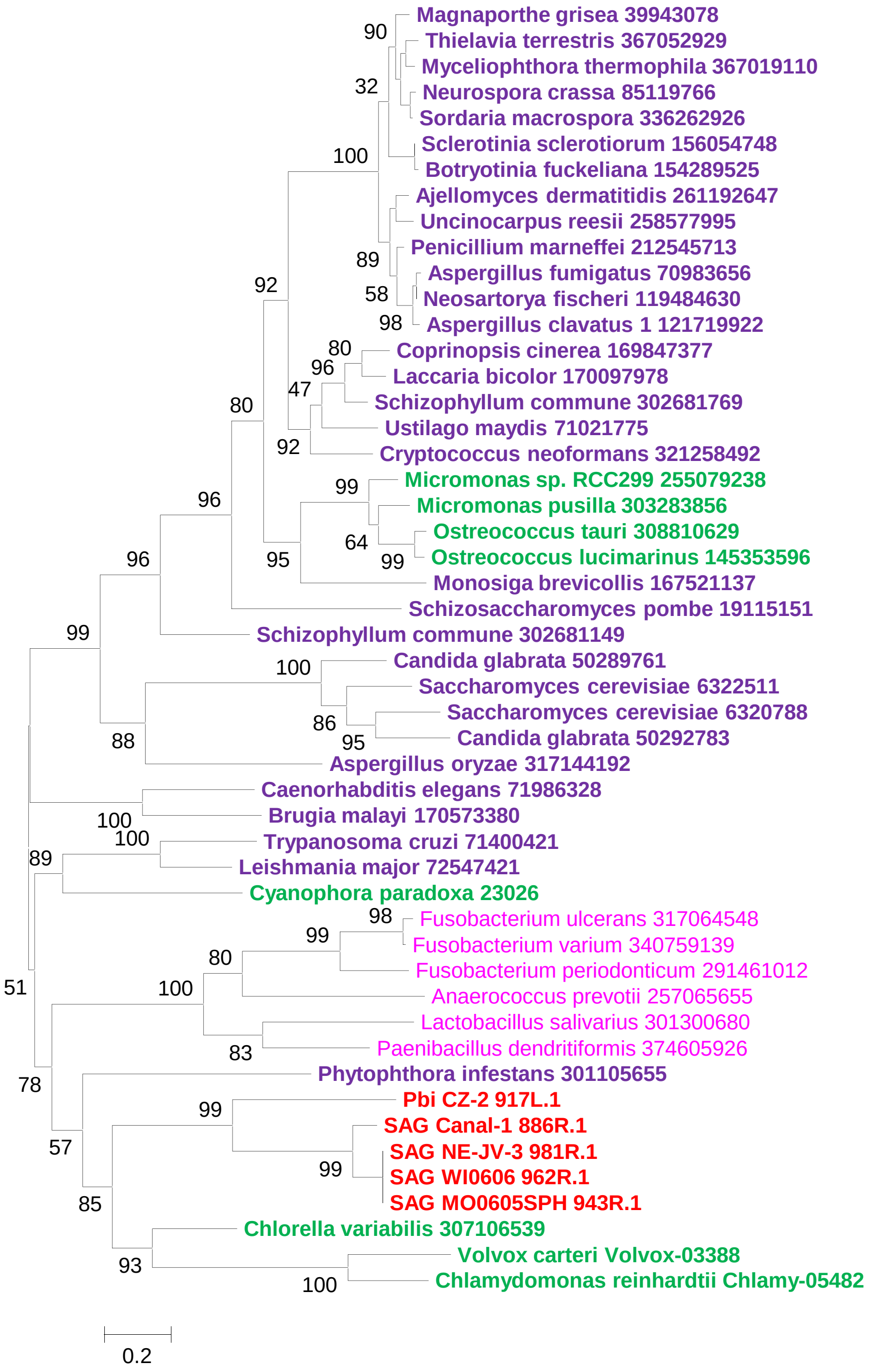

Figure S3: continued

CL0624: THYLAKOID FORMATION 1

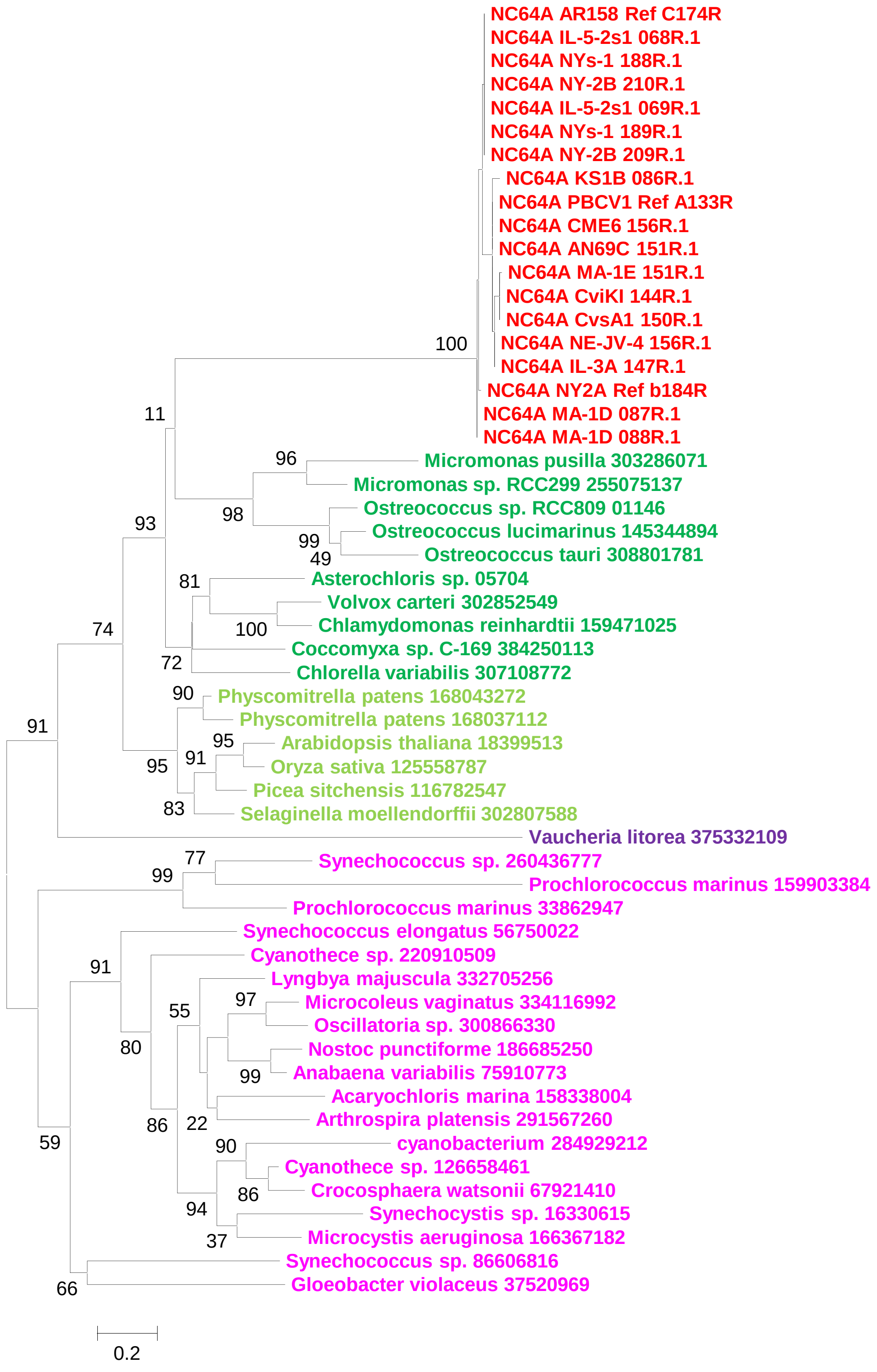

Figure S3: continued

CL0778: unknown function

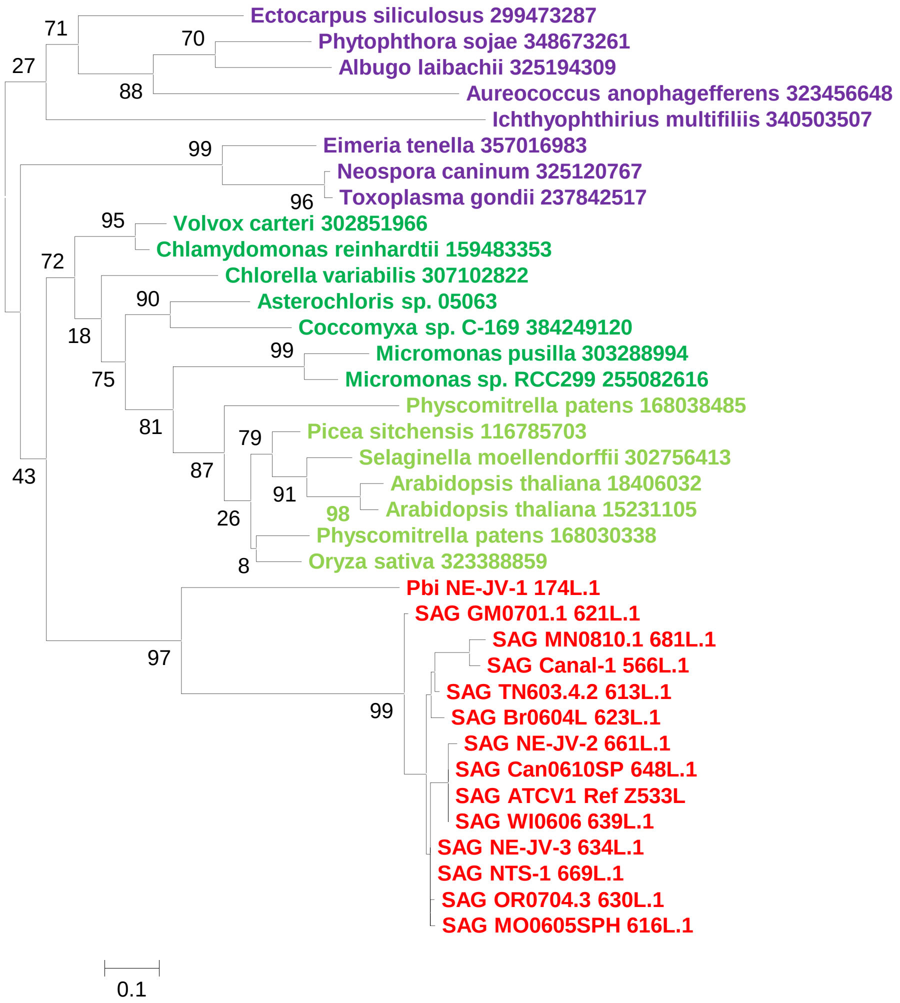

Figure S3: continued

CL0796: ribonucleoside-triphosphate reductase

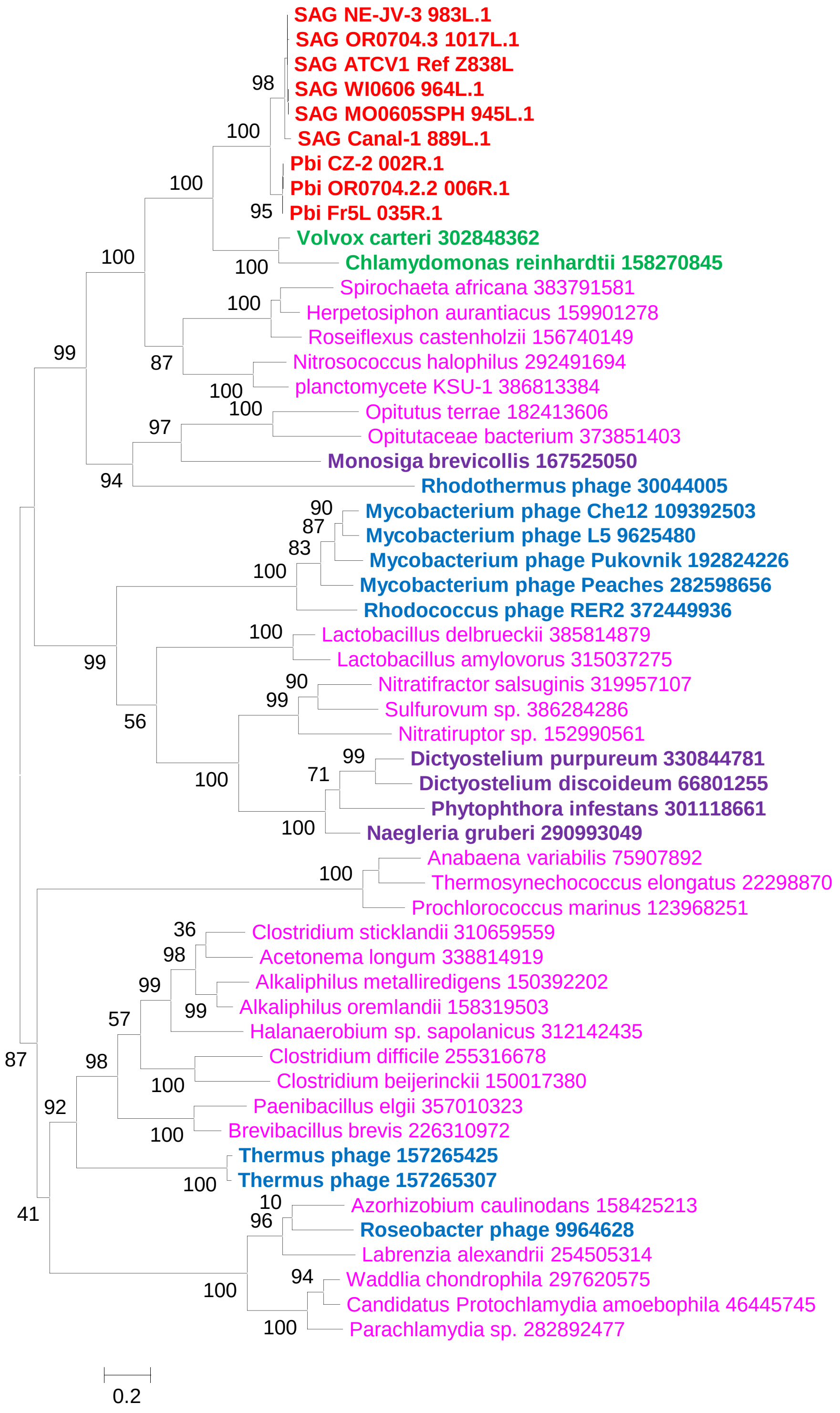

Figure S3: continued

CL0063: unknown function

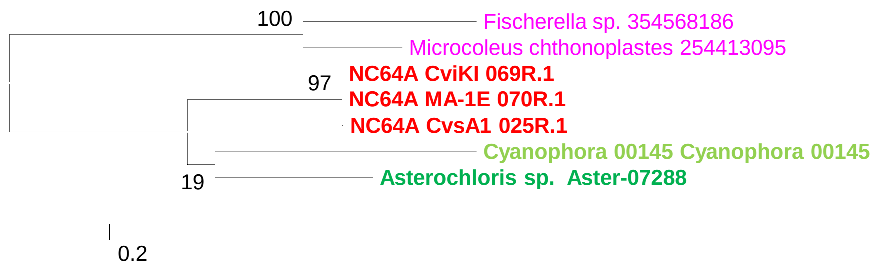

CL0978: unknown function

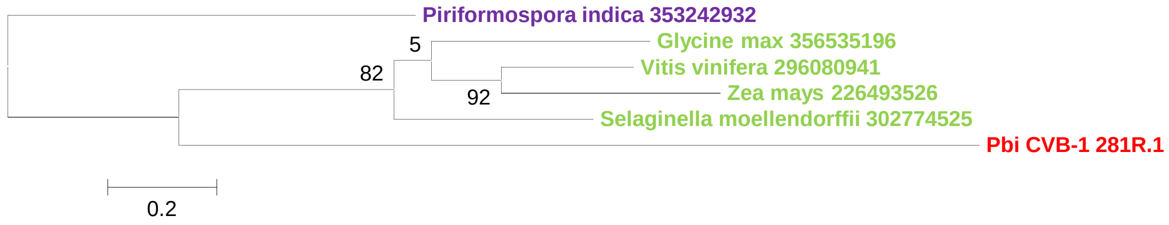

CL0375: glycosyltransferase

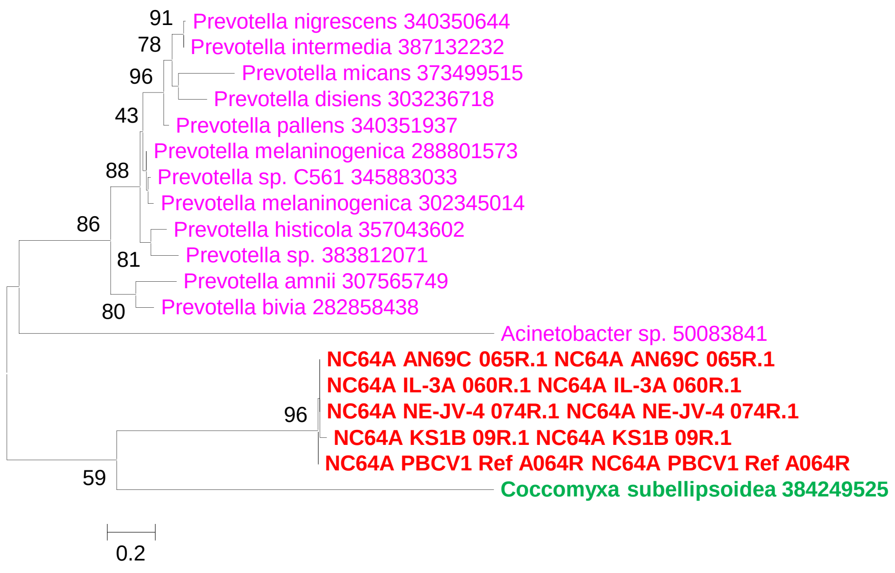

Figure S3: continued

CL0940: Nitroreductase

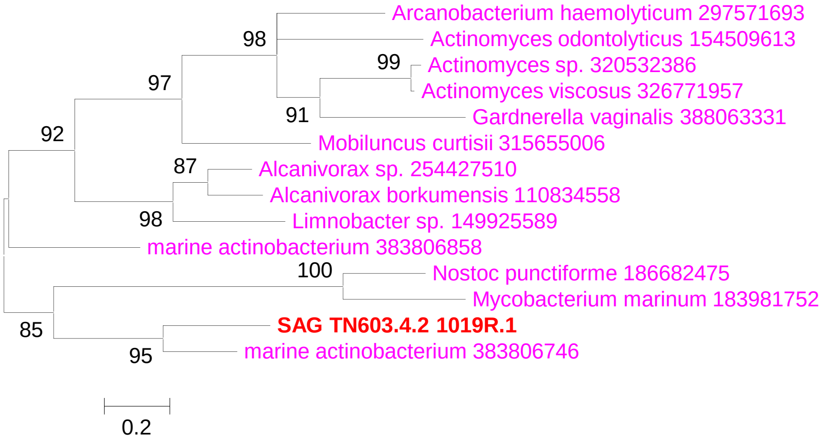

CL0065: unknown function

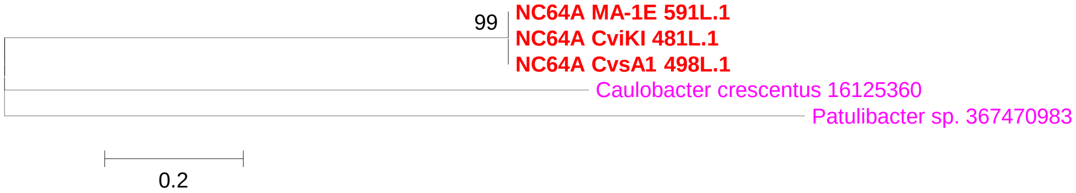

CL0879: unknown function

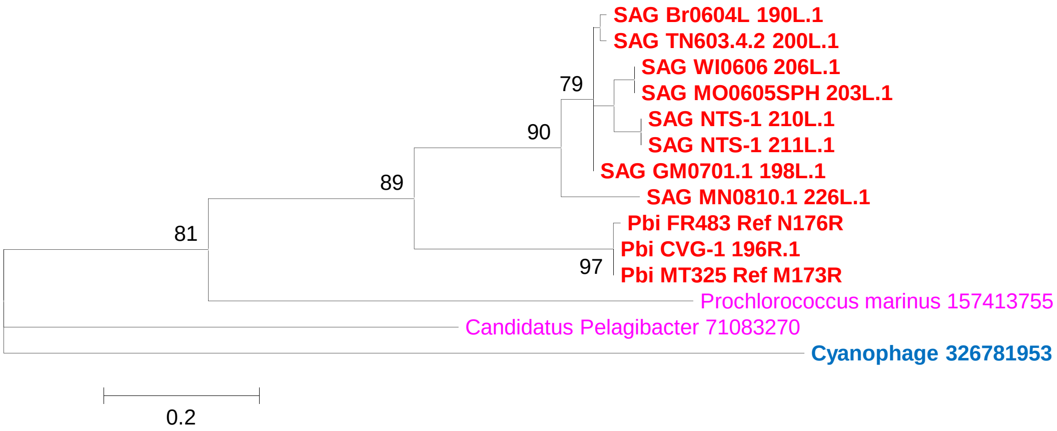

Figure S3: continued

CL0878: dTDP-glucose pyrophosphorylase /HAD superfamily hydrolase

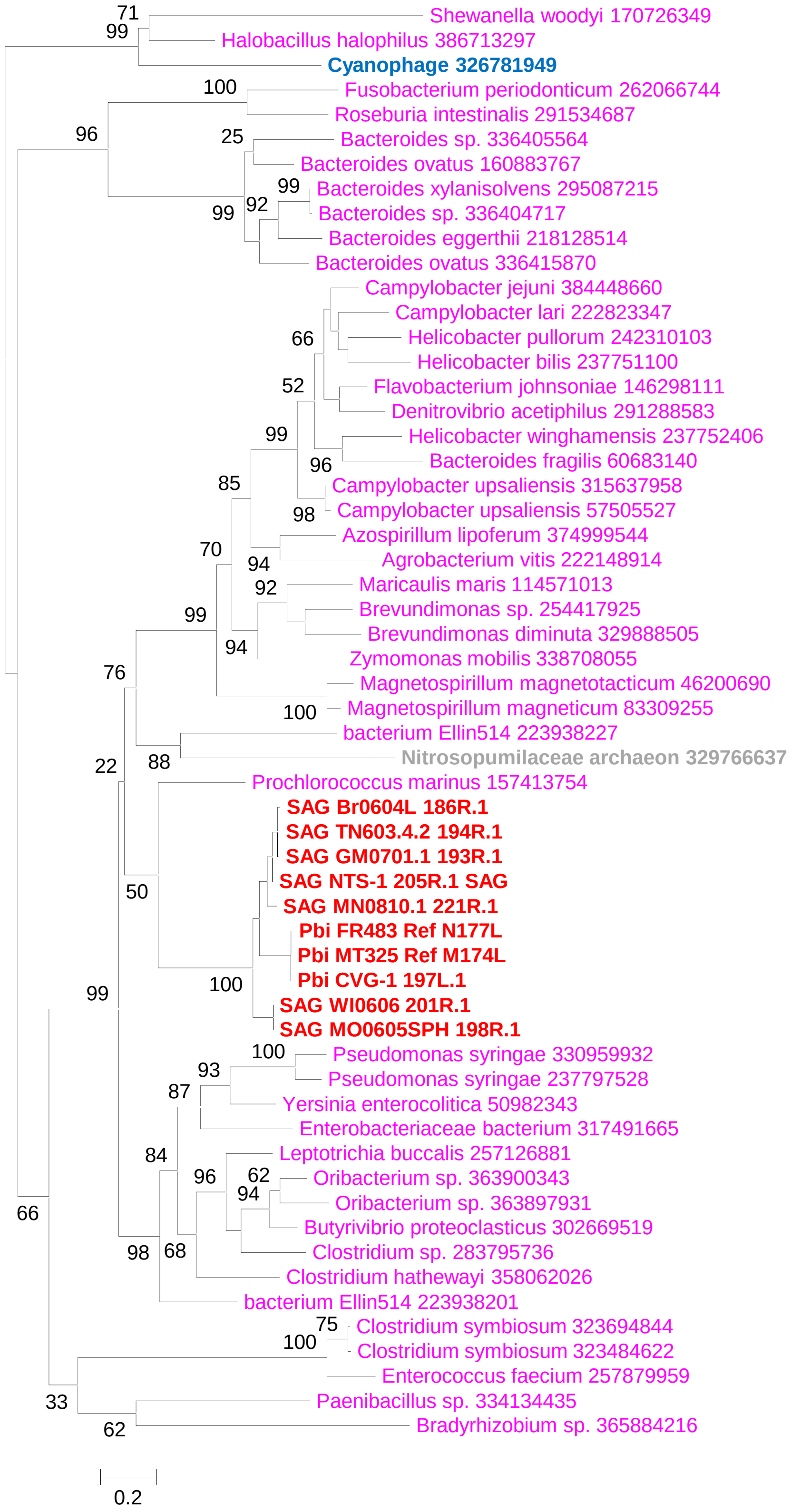

Figure S3: continued

CL0222: DNA methylase

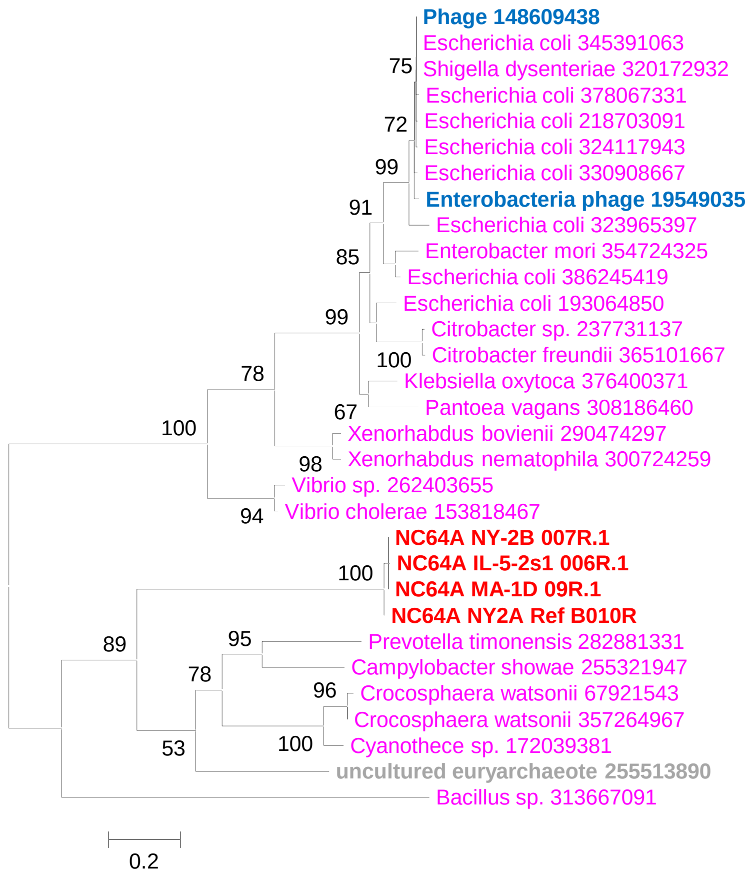

CL0792: mannose-6-phosphate isomerase

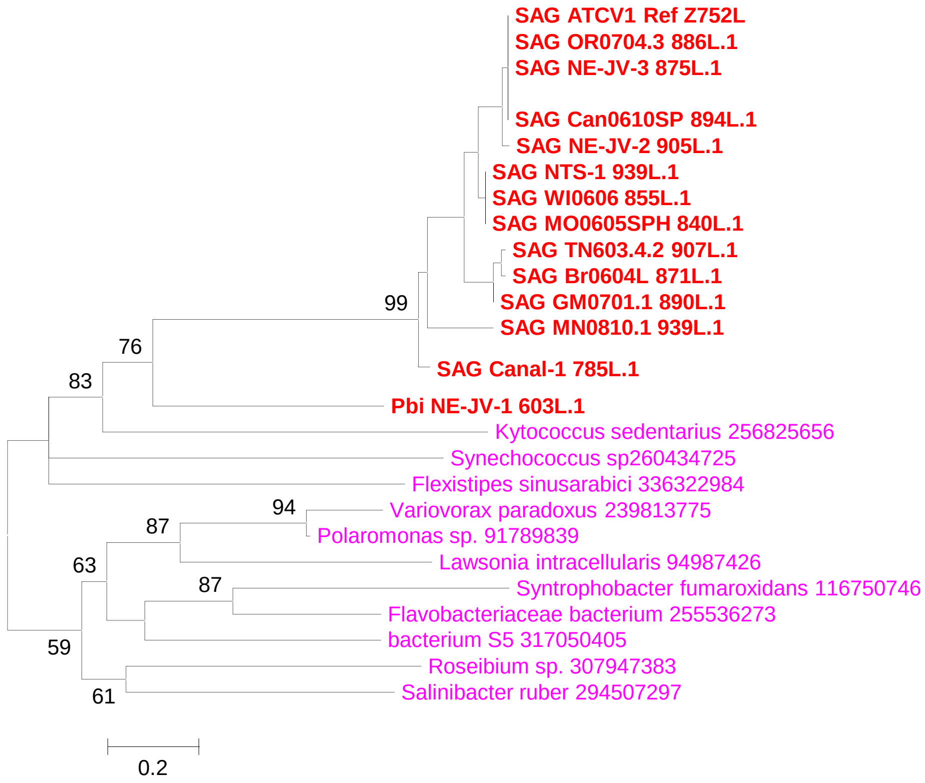

Figure S3: continued

CL0482: glutaredoxin-like

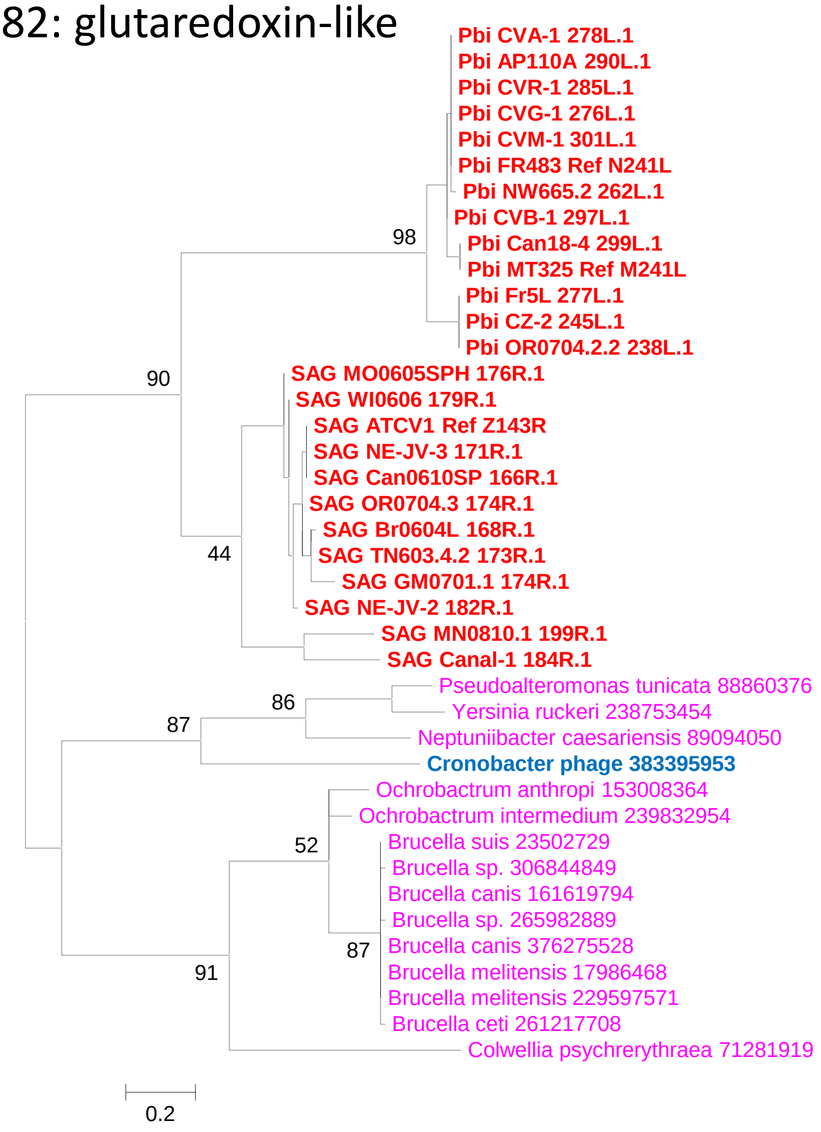

CL0055: unknown function

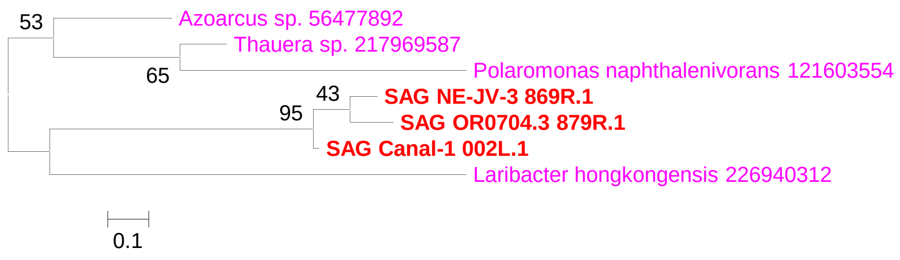

Figure S3: continued

CL0739: aspartate carbamoyltransferase

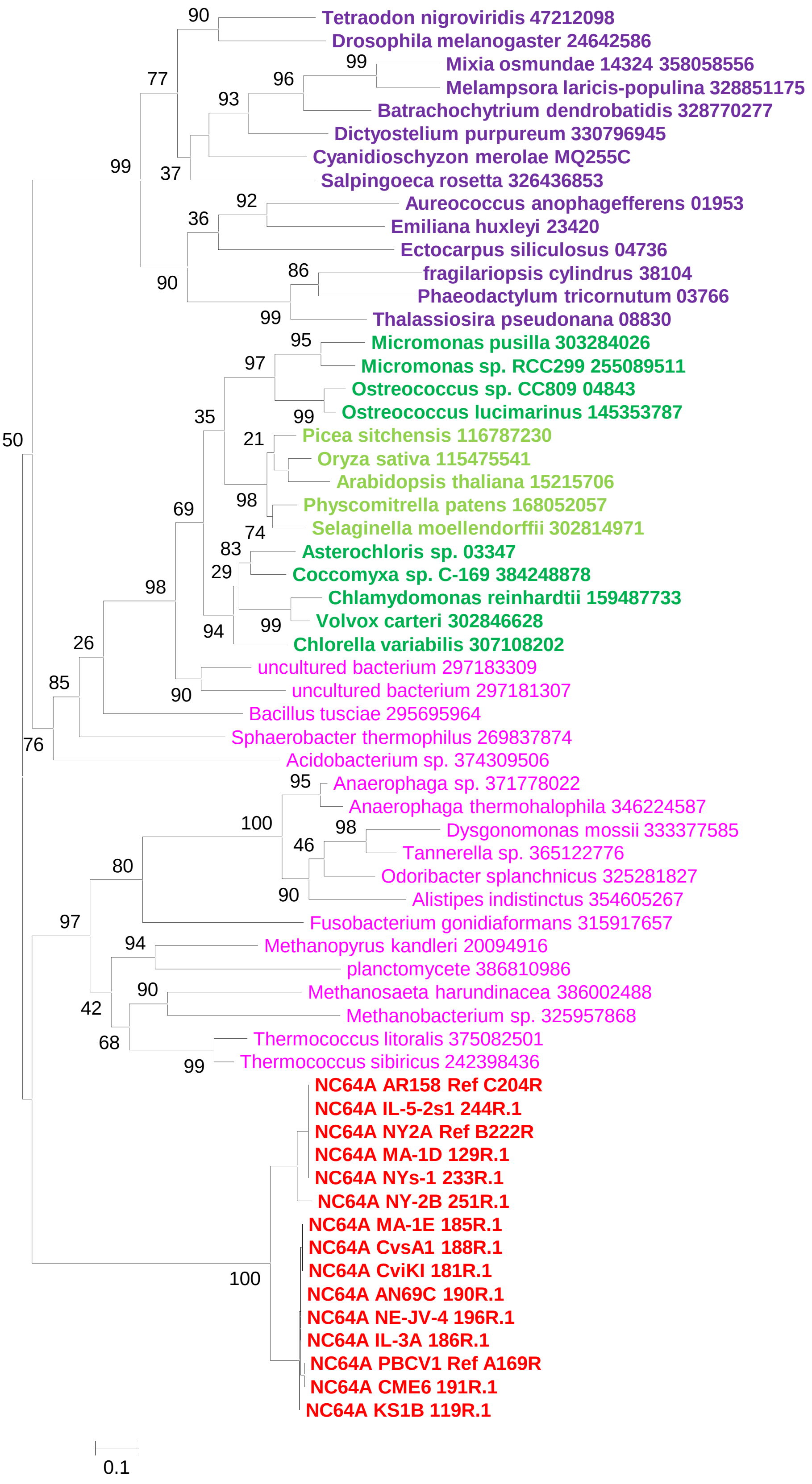

Figure S3: continued

CL0787:Potassium transporter

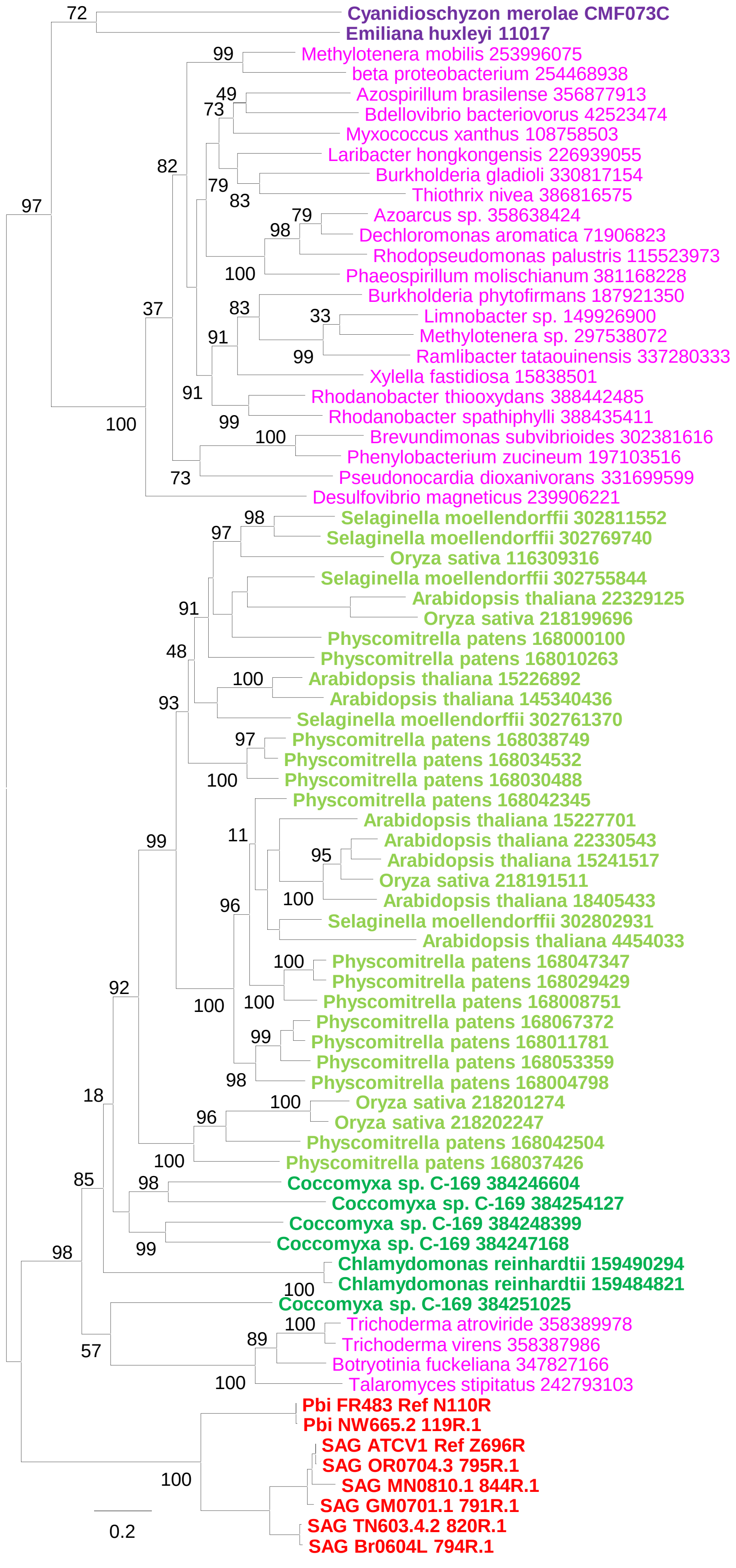

Figure S3: continued

CL0989: unknown function

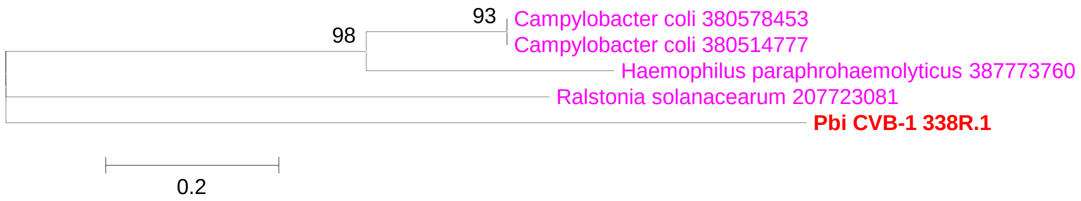

CL0767: unknown function

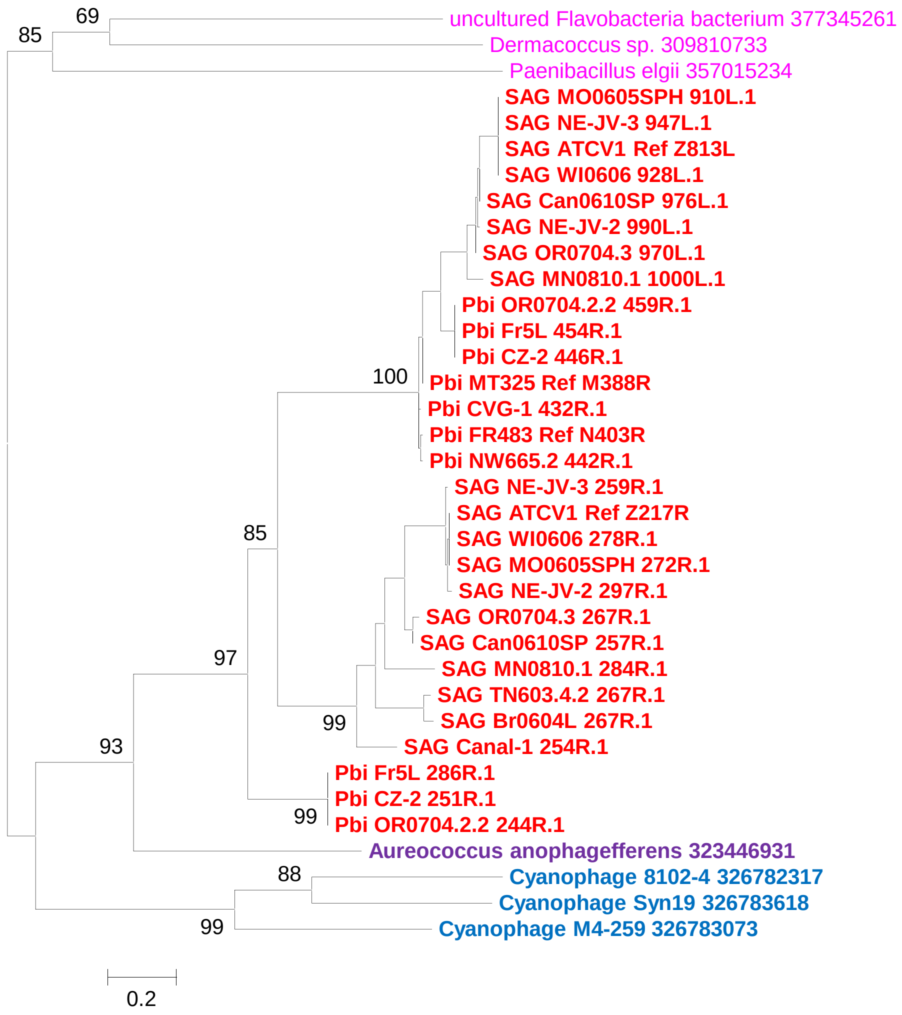

Figure S3: continued

CL0037: beta-lactamase-like

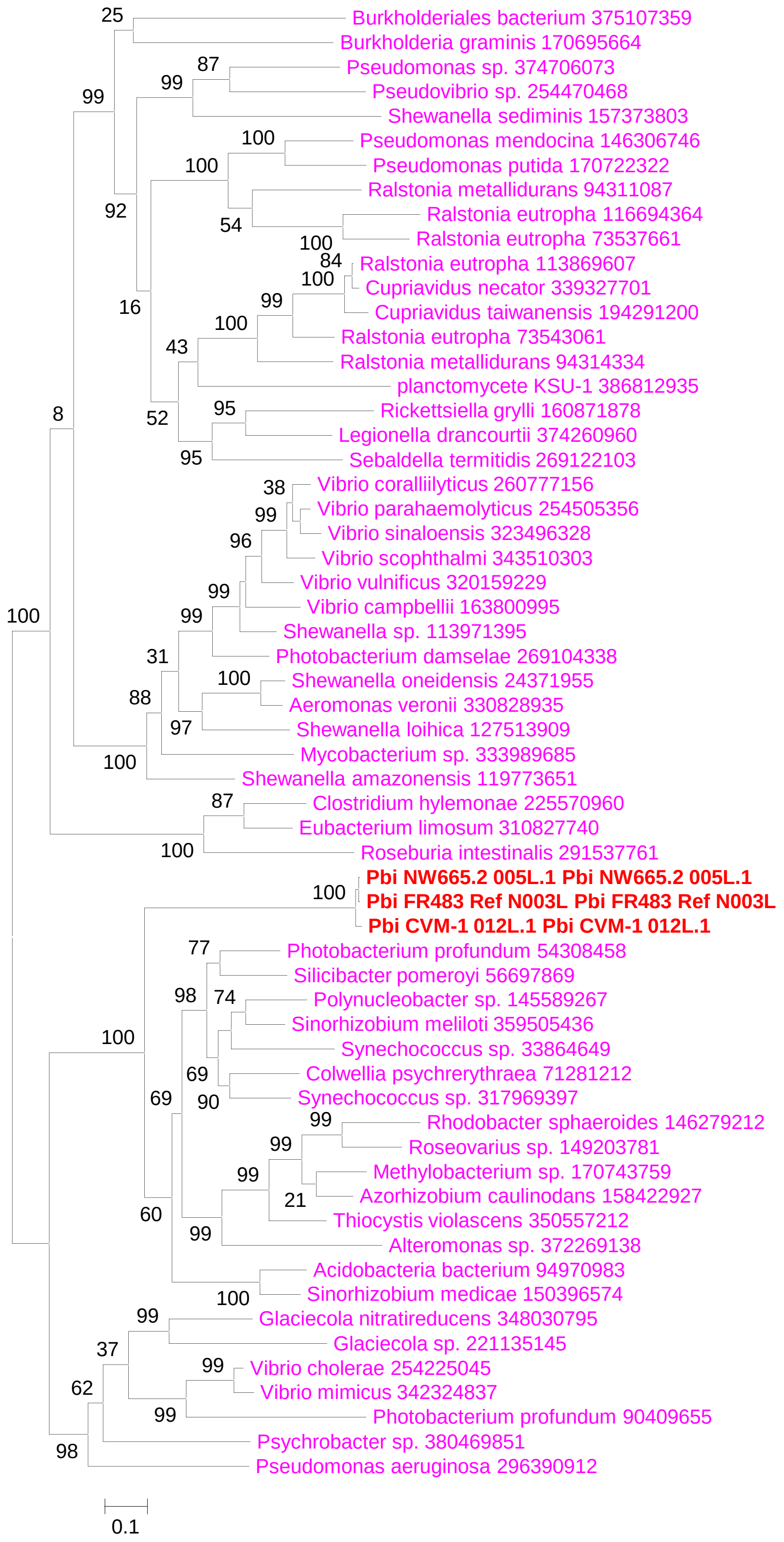

Figure S3: continued

CL0561: Glycosyltransferase family 17

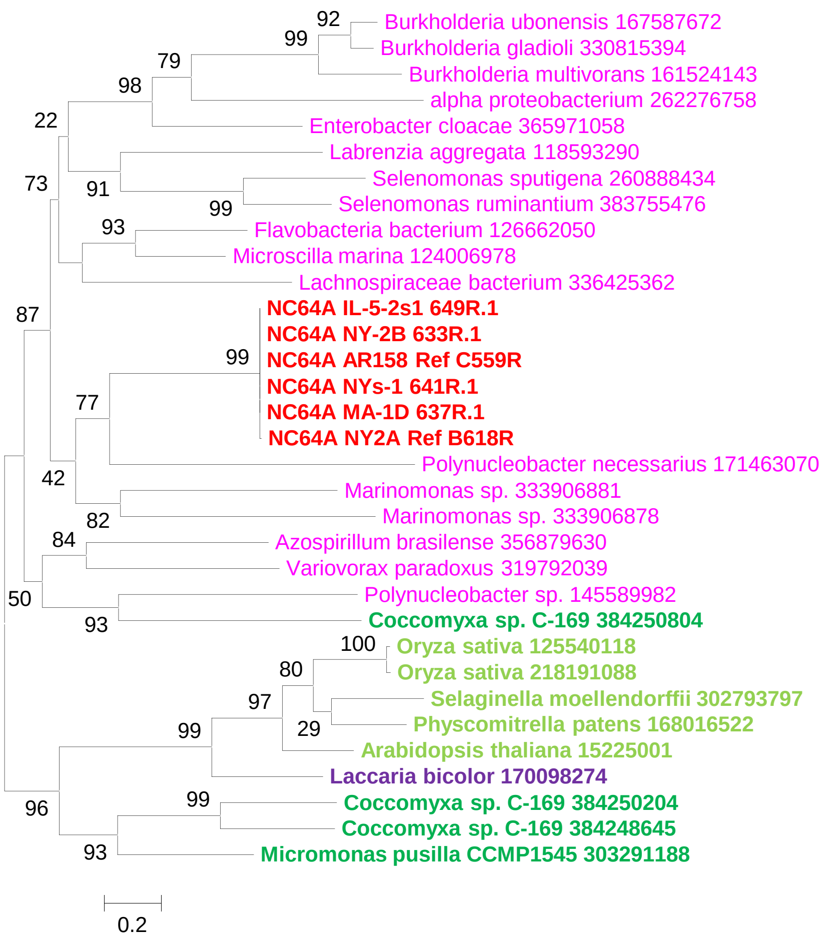

CL0876: methyltransferase

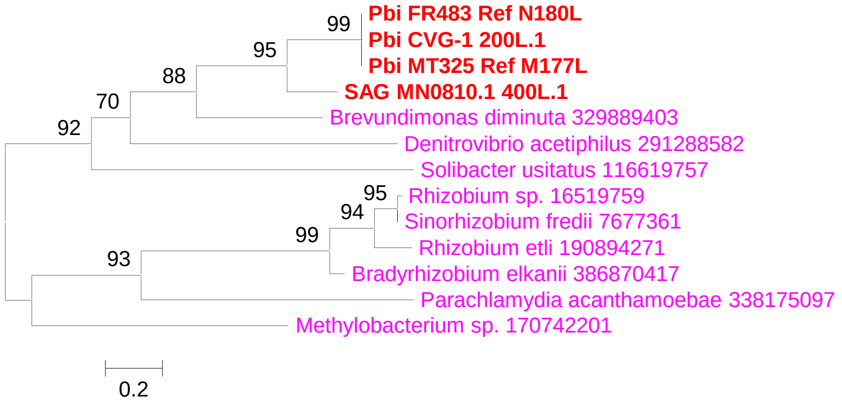

Figure S3: continued

CL0356: methyltransferase

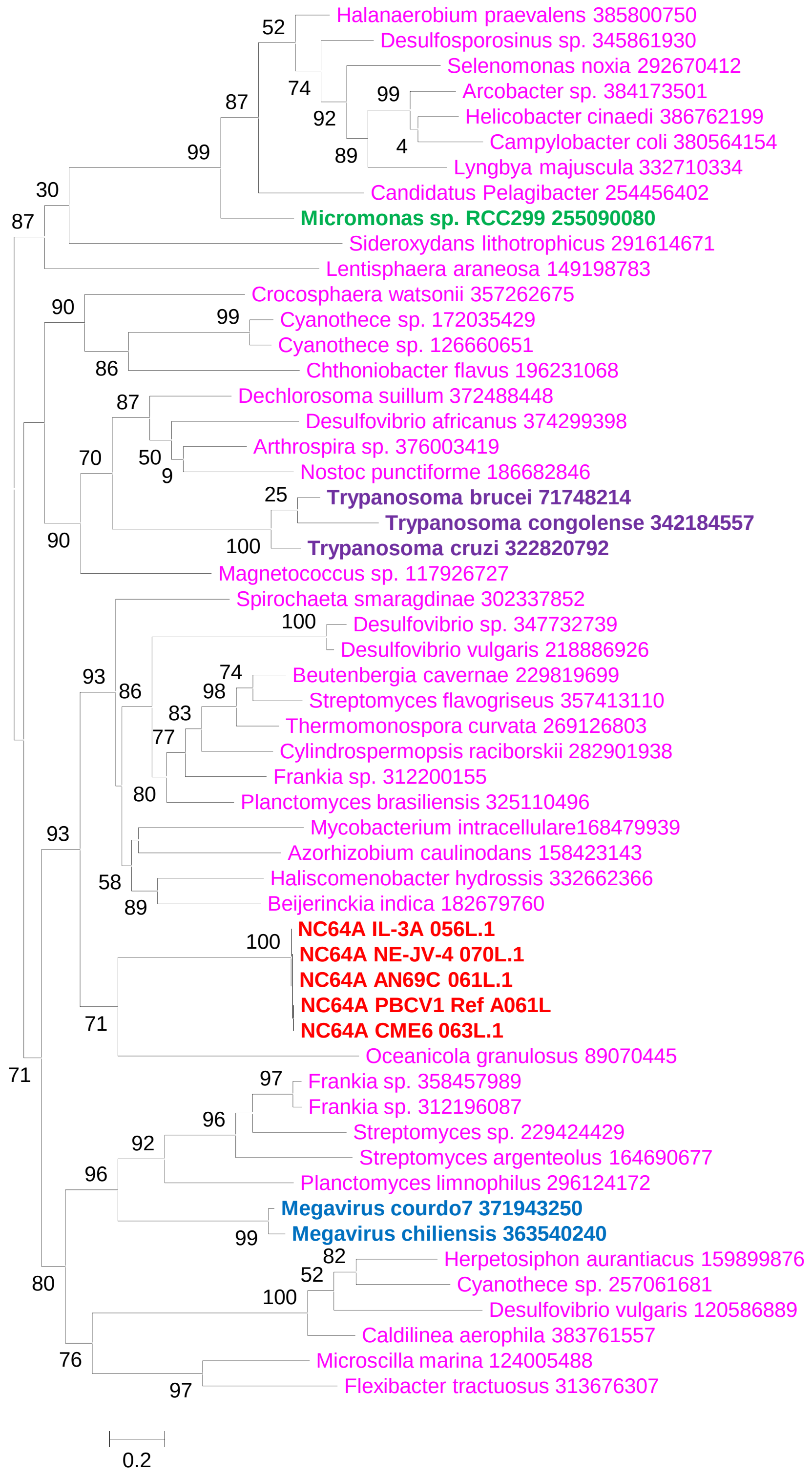

Figure S3: continued

CL0607: methyltransferase

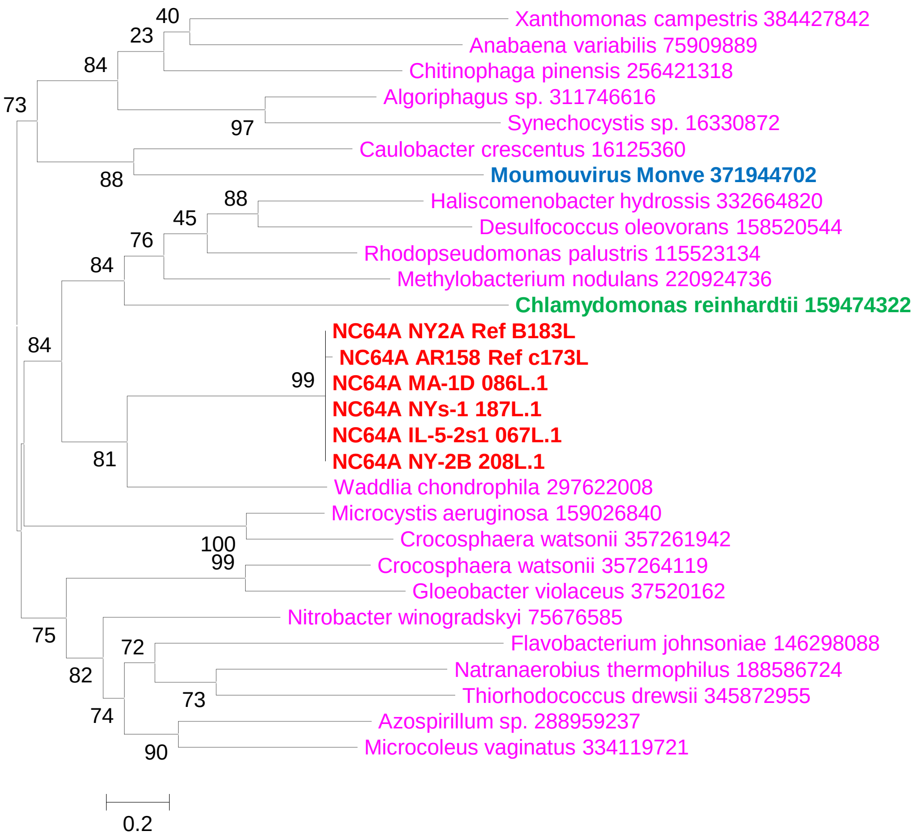

CL0533: unknown function

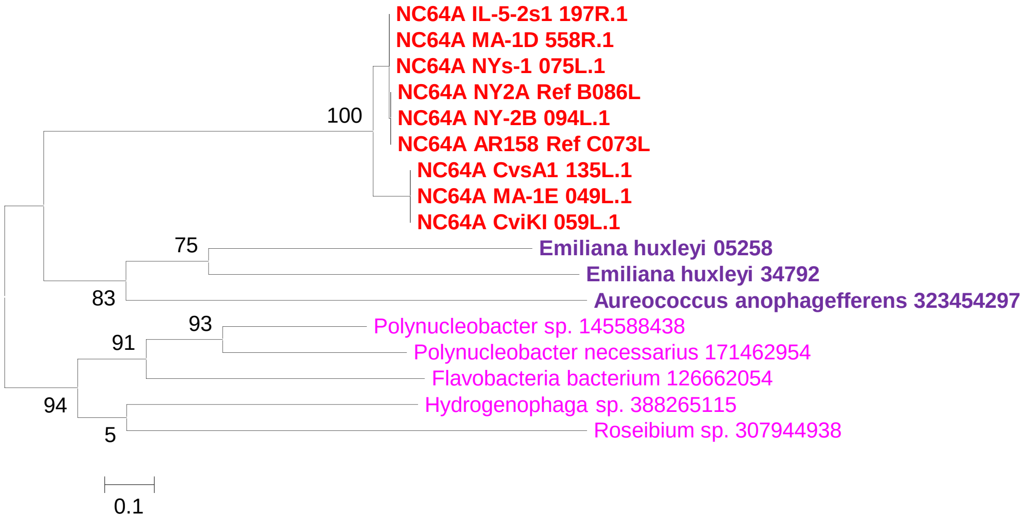

Figure S3: continued

CL0007: ADP-ribosyl glycohydrolase

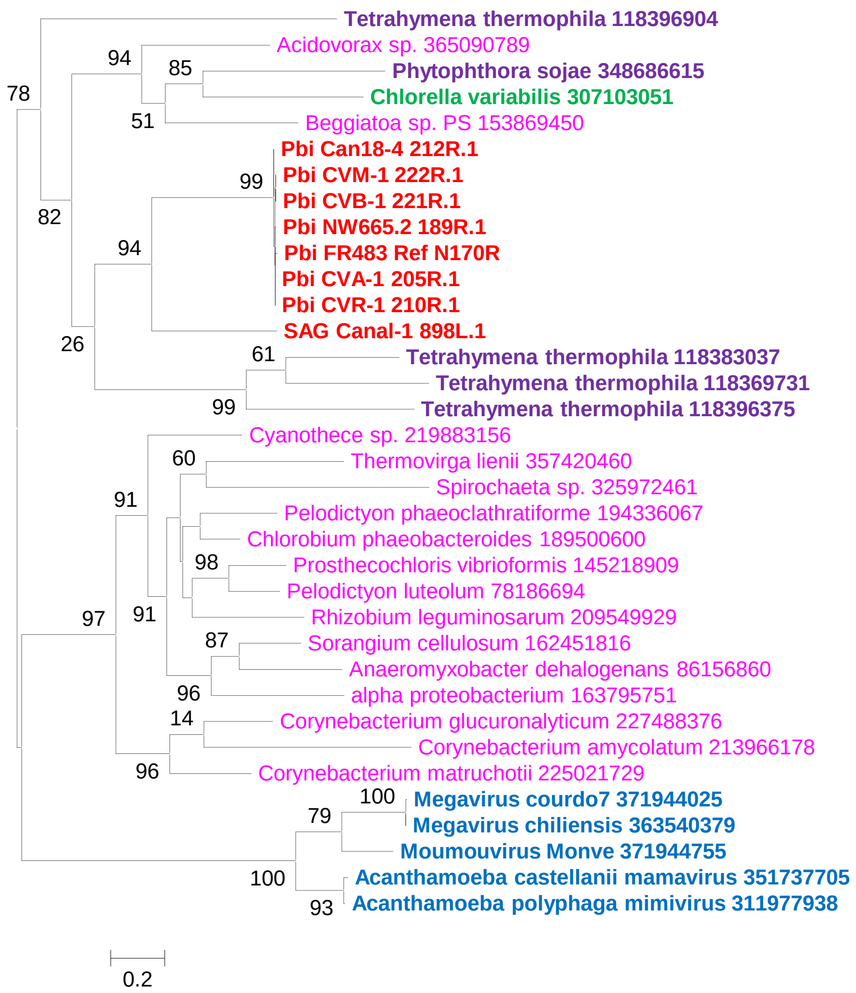

CL0875: methyltransferase

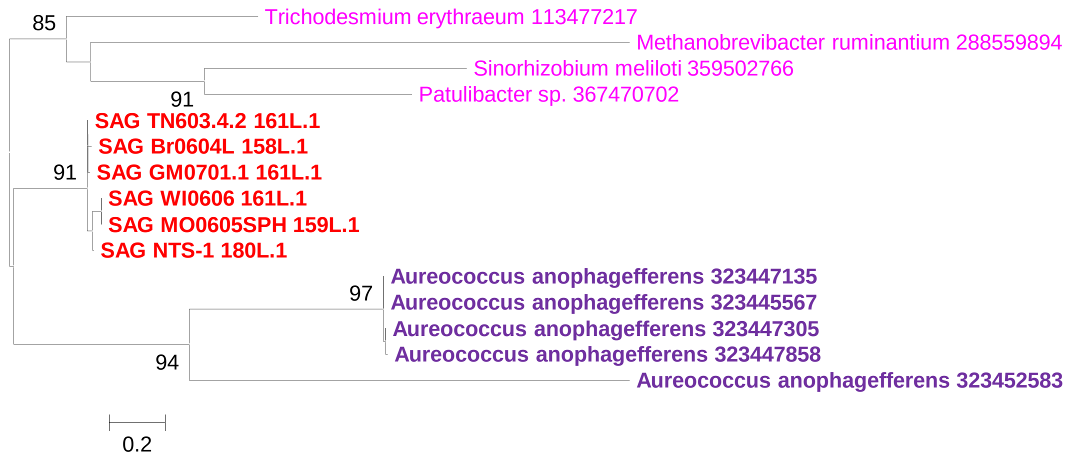

Figure S3: continued

CL0489: unknown function

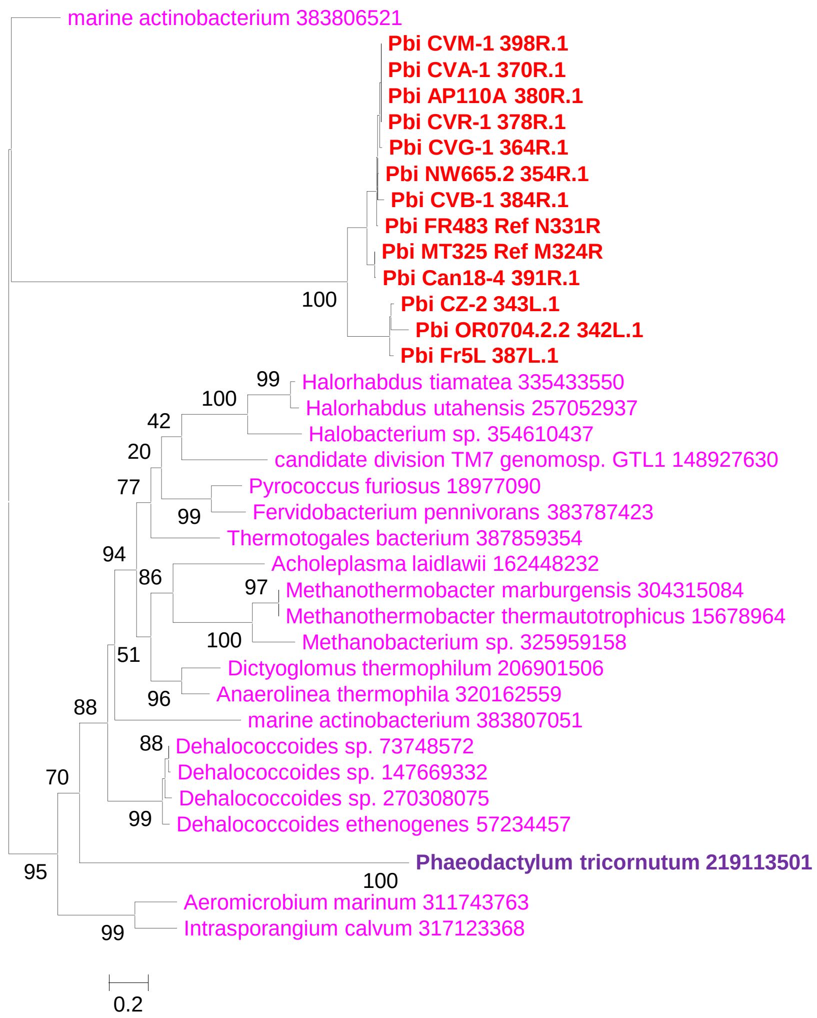

CL0963: glycosyl hydrolase

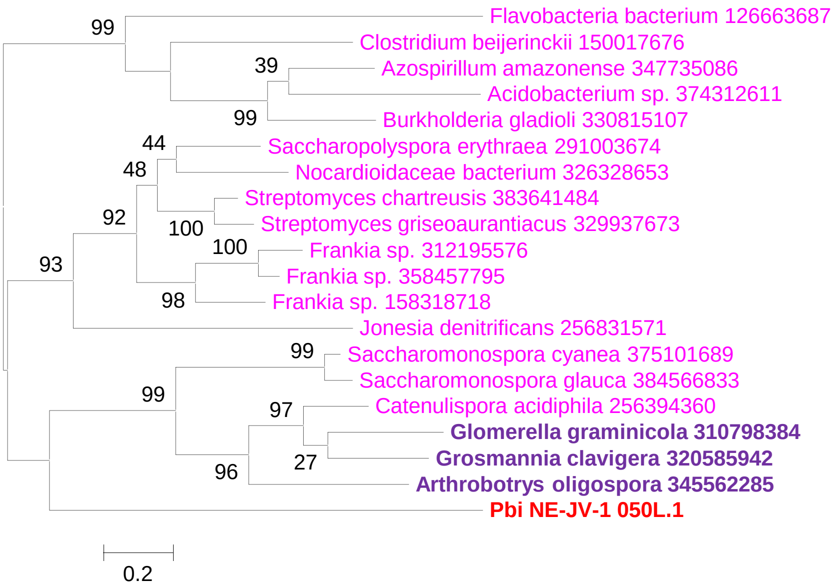

Figure S3: continued

CL0957: NADH-dependent oxidoreductase

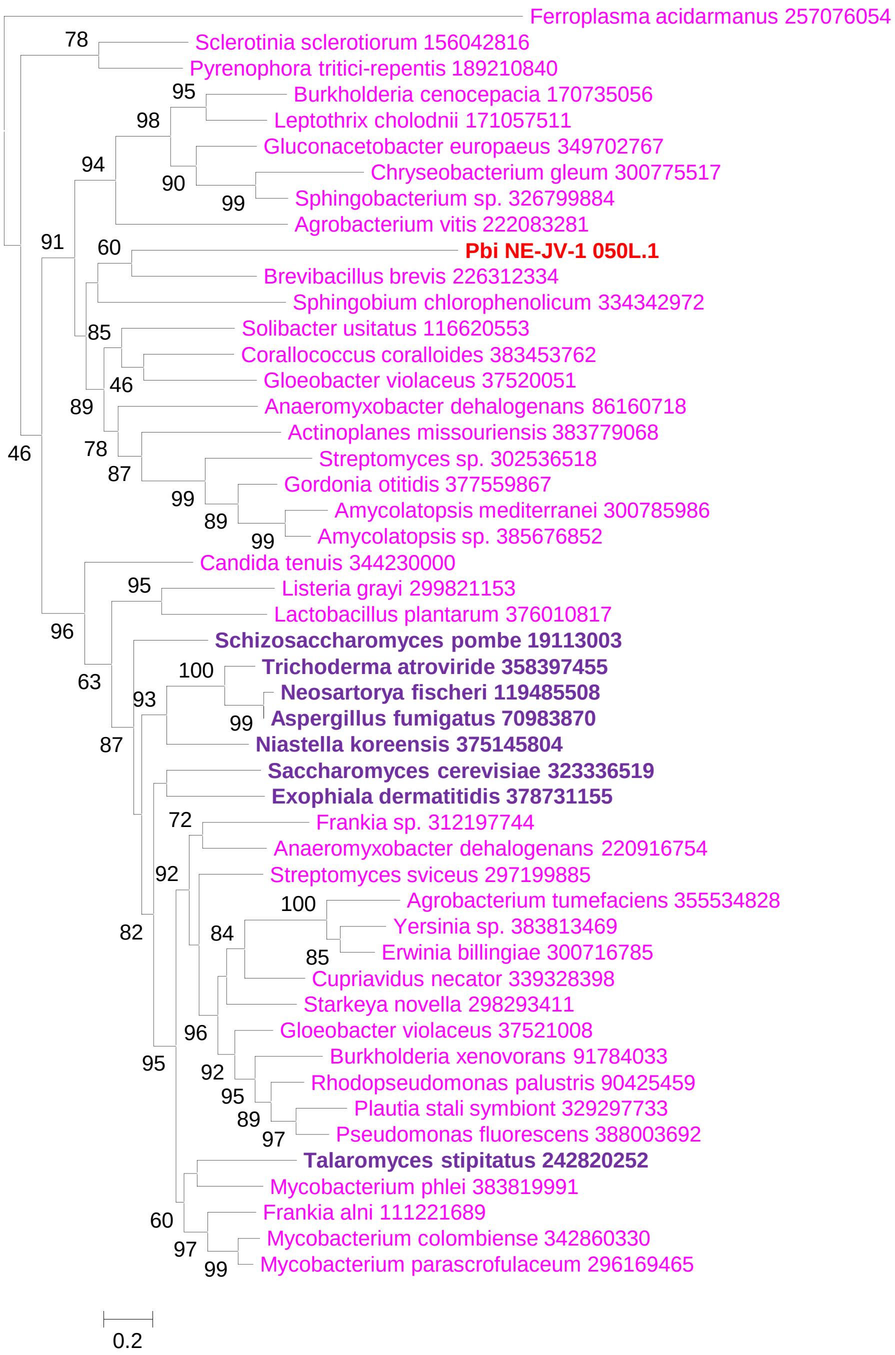

Figure S3: continued

CL0011: unkwown function

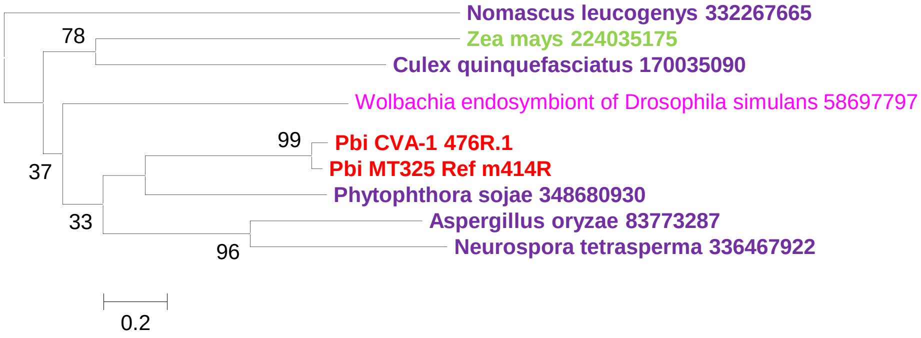

CL0056: unknown function

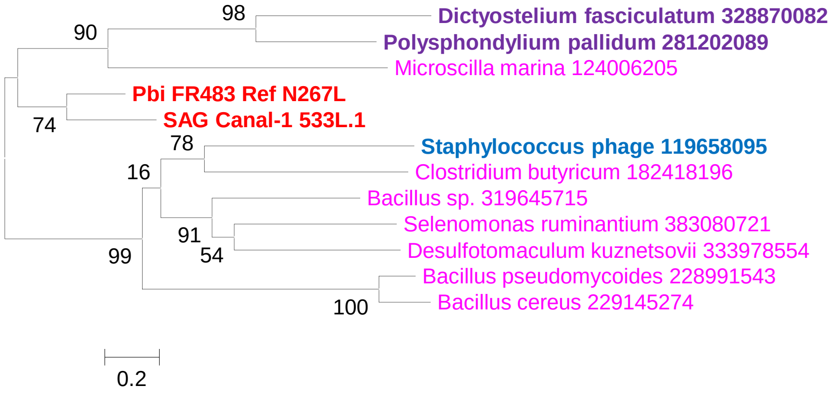

CL0503: unknown function

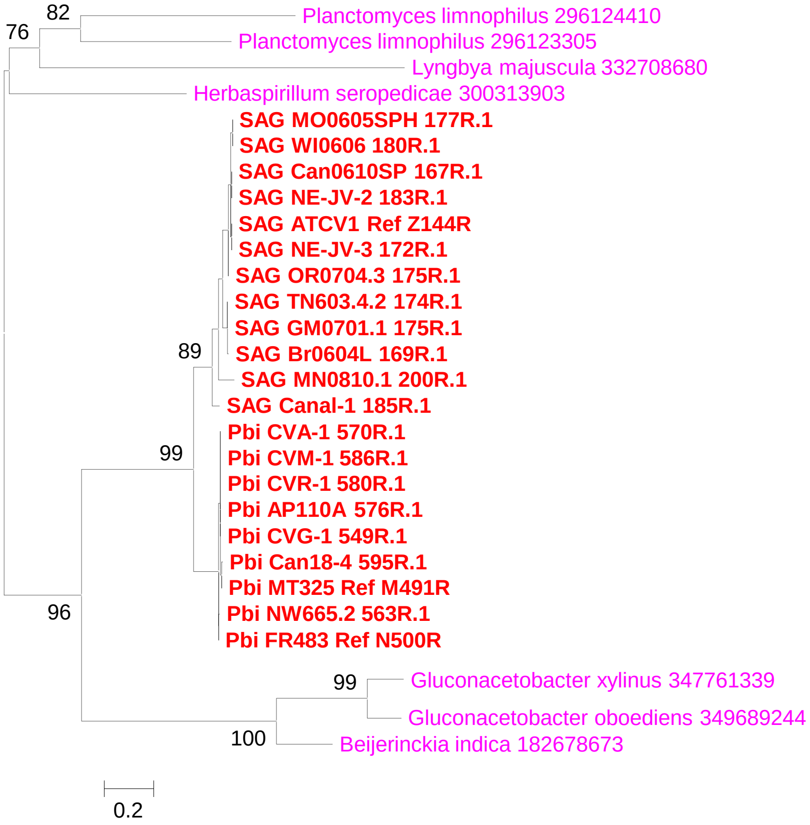

Figure S3: continued
