# Supplementary material for: Towards defining the chloroviruses: a genomic journey through a genus of large DNA viruses
Source: BMC Genomics. 2013 Mar 8;14:158. doi: 10.1186/1471-2164-14-158 (PMC3602175; doi:10.1186/1471-2164-14-158)
Supplement: Additional file 8: Figure S4. — Phylogenetic trees showing potential HGT between chloroviruses and Chlorella. Trees were reconstructed using the ML method using the WAG + G + I substitution model. Interior branch support was estimated by the approximate likelihood ratio test (aLRT). For the sake of clarity, we only show branch support for important clades. Taxon names are colorized according to taxonomic information: Chlorovirus (red), chlorophytes (dark green), streptophytes (light green), eukaryote (violet), prokaryote (pink) and DNA virus (blue). Genbank gi numbers are given after species names. Protein family ID and functional annotation are given above each tree. [file 1471-2164-14-158-S8.pdf]

Figure S4

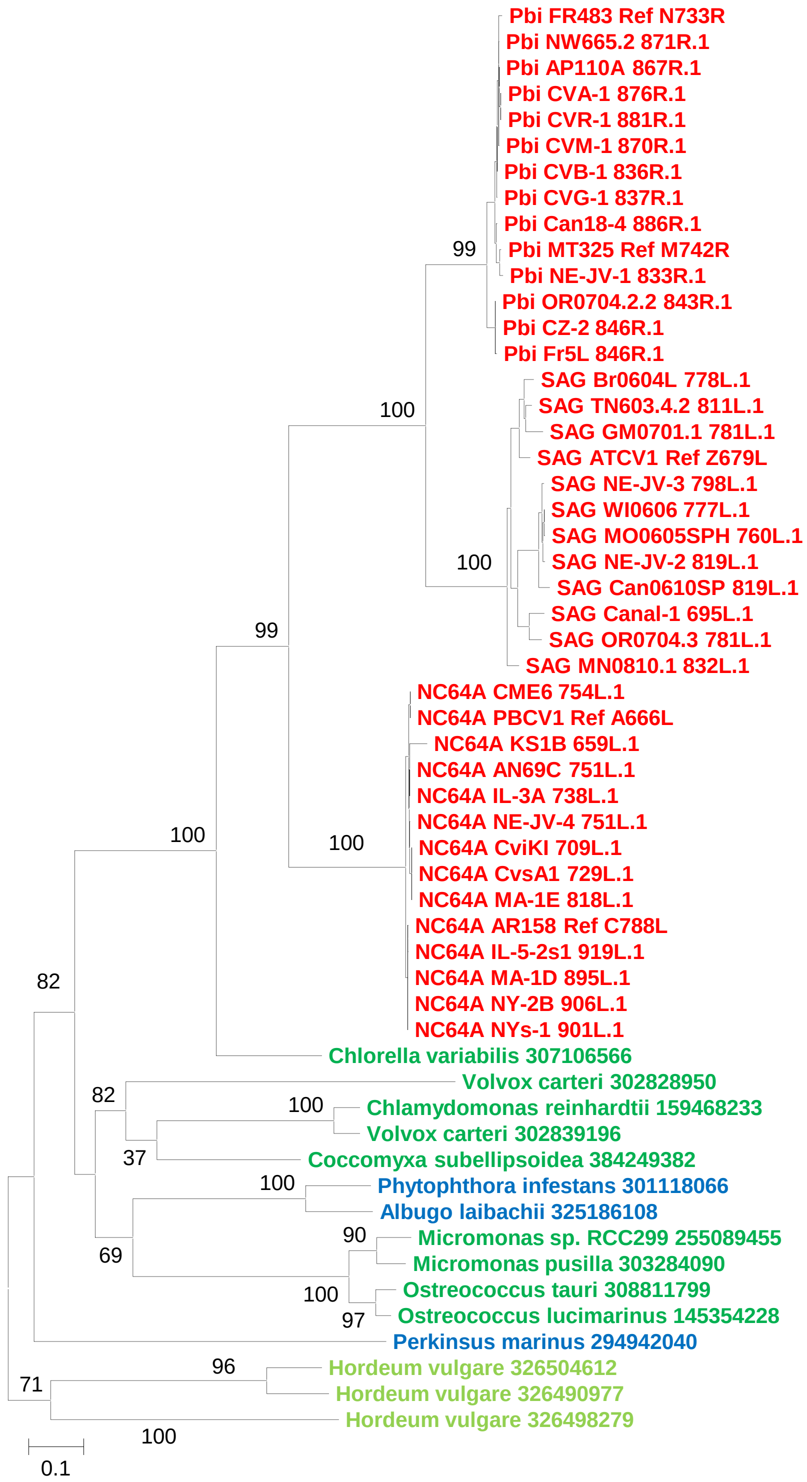

CL0450: translation elongation factor ElF3

CL0511: unknown function

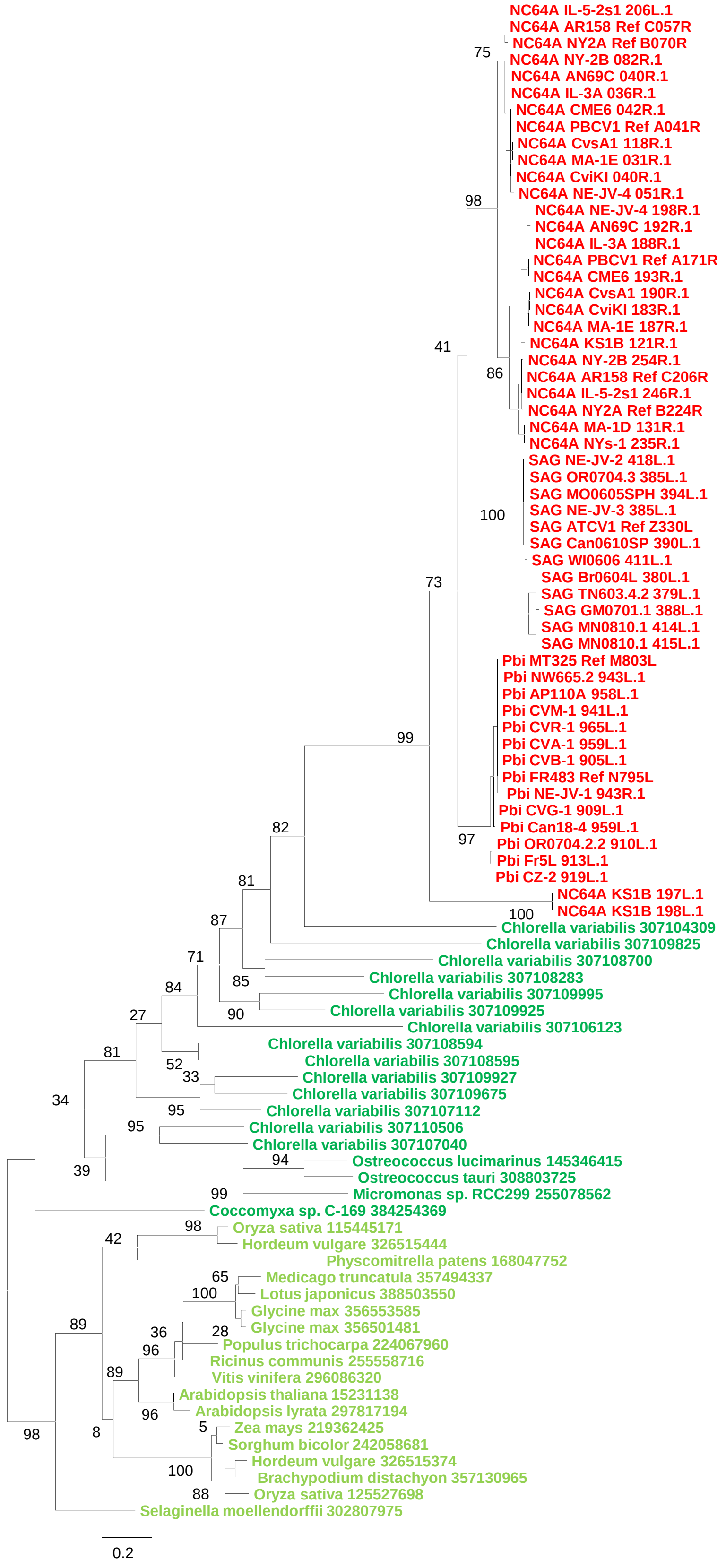

Figure S4: continued

CL0288: chitin deacetylase

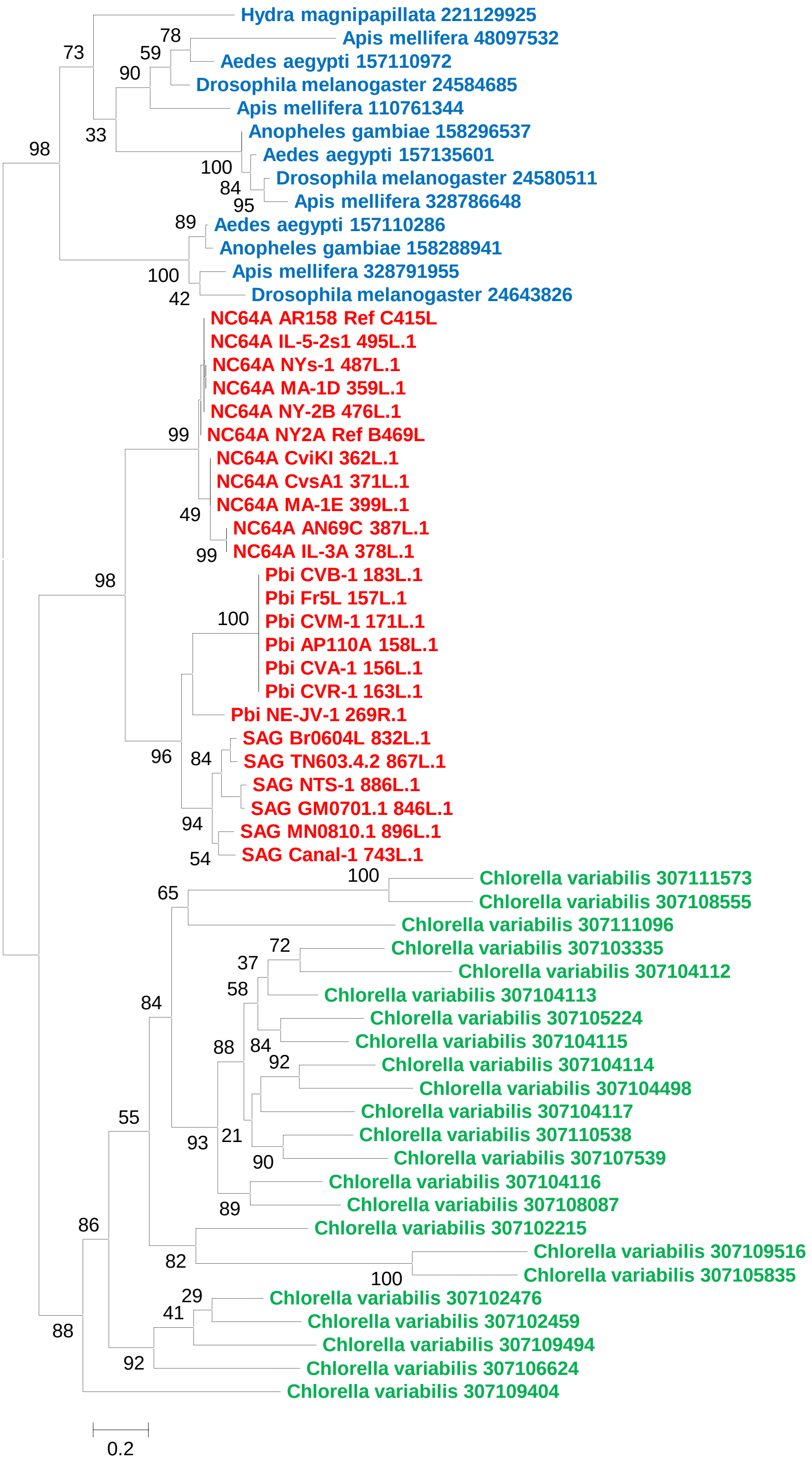

Figure S4: continued

CN0022: unknown function

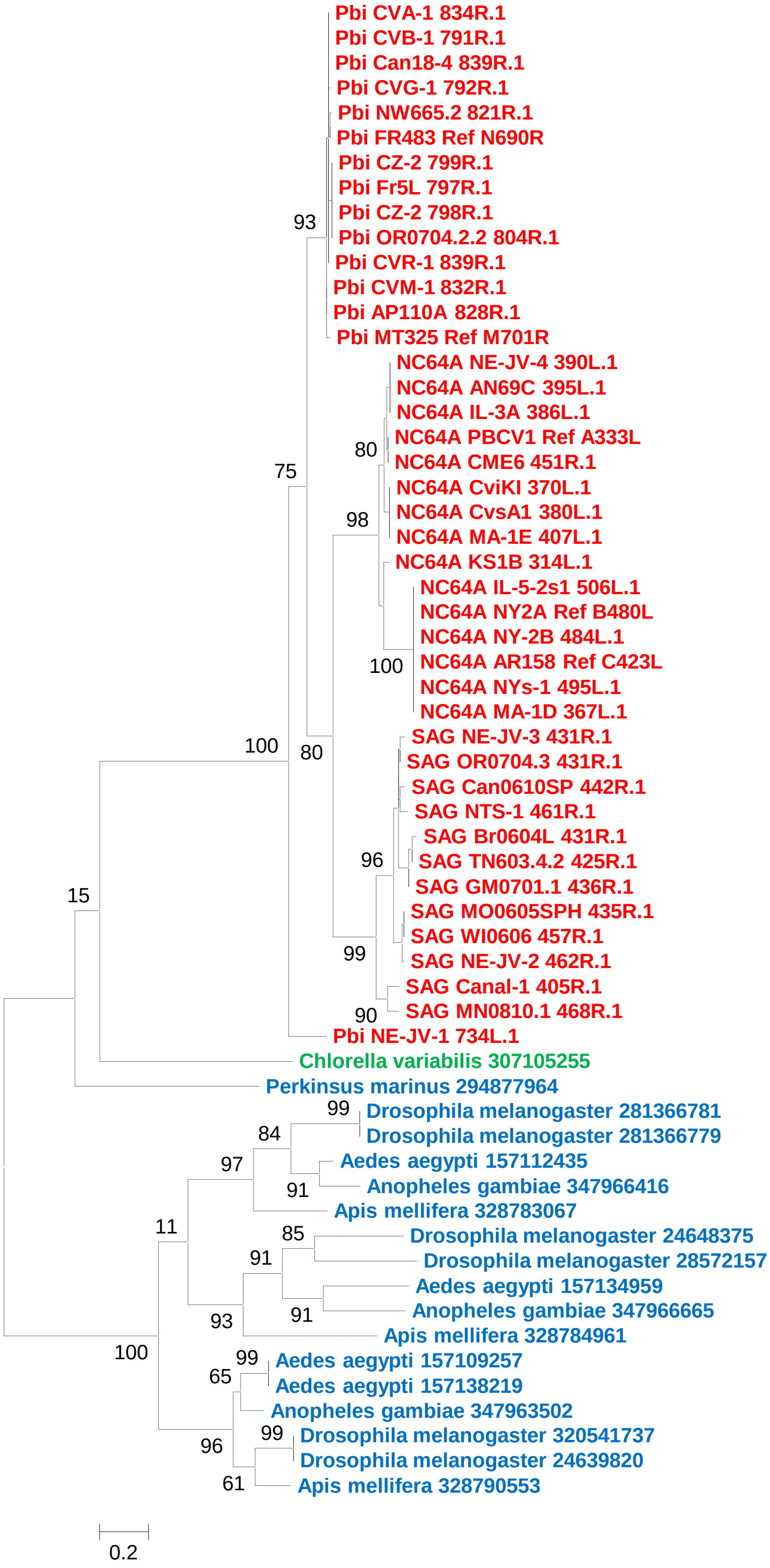

Figure S4: continued

CL0887: chitinase

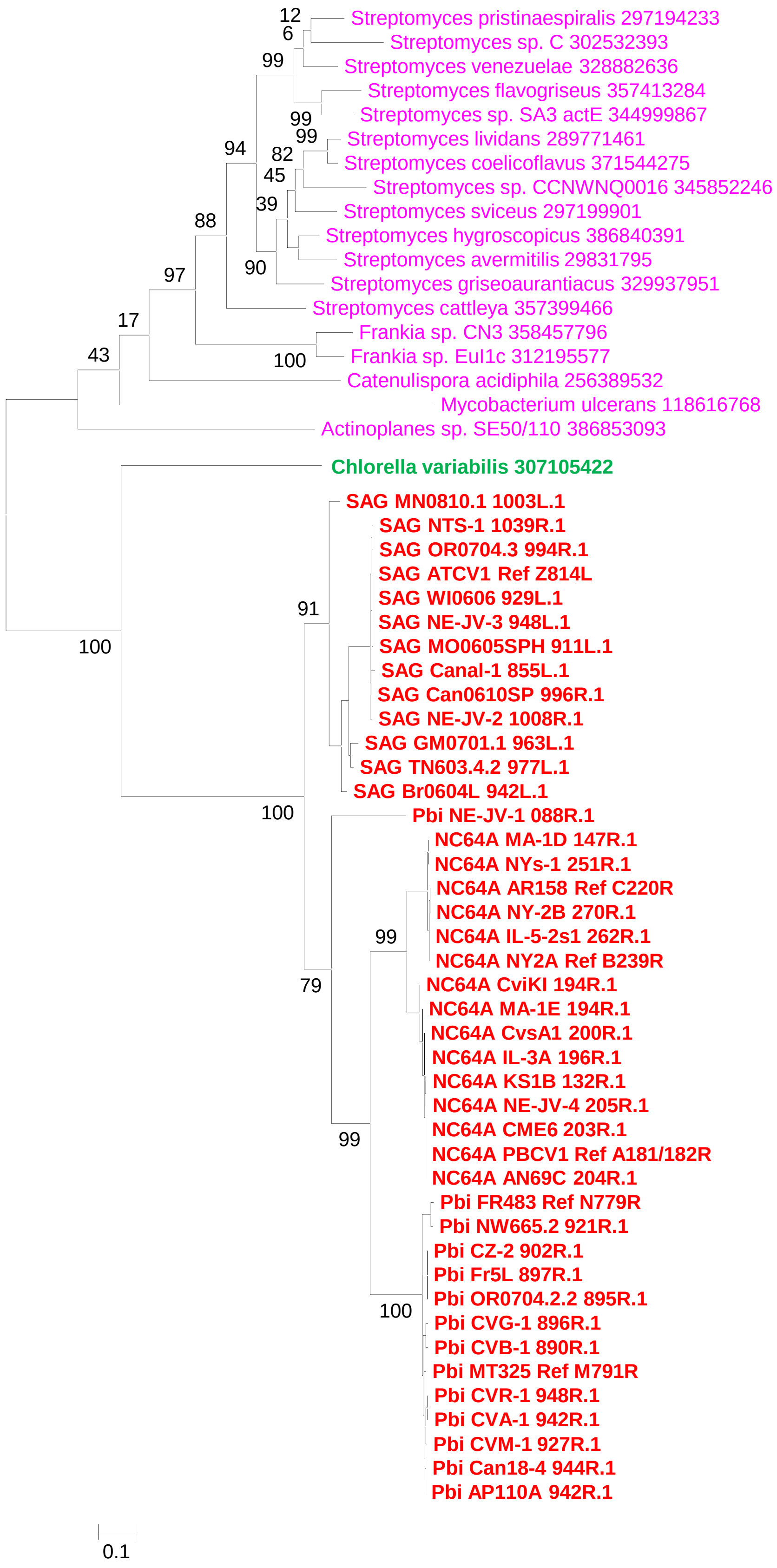

Figure S4: continued

CL0531: unknown function

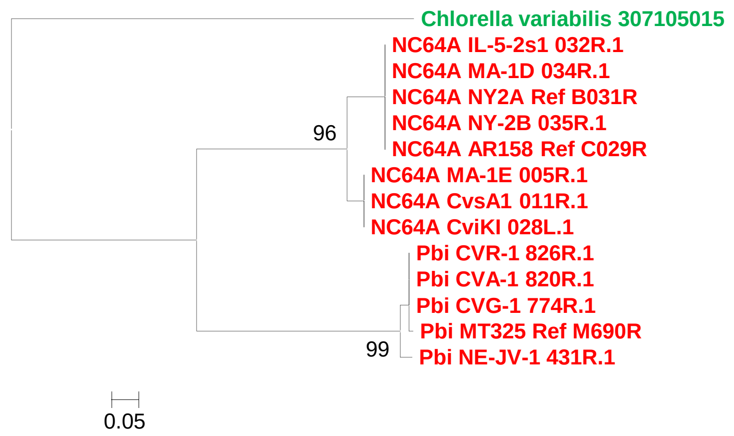

Figure S4: continued
