# Supplementary material for: Towards defining the chloroviruses: a genomic journey through a genus of large DNA viruses
Source: BMC Genomics. 2013 Mar 8;14:158. doi: 10.1186/1471-2164-14-158 (PMC3602175; doi:10.1186/1471-2164-14-158)
Supplement: Additional file 9: Table S5. — Attributes of the sequenced chloroviruses. [file 1471-2164-14-158-S9.pdf]

Table S5: attributes of the sequenced chloroviruses

**Virus**	**Host**	**Attributes and comments (plaque size, plaque morphology, gene content, etc.)**	**Source of isolate**	**Date collected**
Chlorella variabilis NC64A Virus Isolates				
CviKI	Chlorella NC64A	from Yamada Lab, Japan; encodes Hyaluronan Synthetase and Chitin Synthase	Kyoto, Japan	1990
IL-3A	Chlorella NC64A	Serves as the "null mutant" in the hyaluronan/chitin competition series	IL, USA	Oct. 1983
CvsA1	Chlorella NC64A	from Yamada Lab, Japan; encodes 2 Chitin Synthase genes (& gfat)	Sawara, Japan	April 1992
MA-1D	Chlorella NC64A	see [1] table 2; Small plaque-forming virus	MA, USA	Aug. 1984
NYs-1	Chlorella NC64A	see [1] table 2; Small plaque-forming virus	NY, USA (river)	Aug. 1985
IL-5-2s1	Chlorella NC64A	see [1] table 2; Small plaque-forming virus	IL, USA (farm pond)	May 1986
KS1B	Chlorella NC64A	Small plaque-forming virus	Kansas, USA	May 2003
NY-2B	Chlorella NC64A	see [1] table 2; Very small plaque-forming virus	NY, USA (river)	Aug. 1984
AN69C	Chlorella NC64A	Small plaque-forming virus	Canberra, Australia	March 1995
MA-1E	Chlorella NC64A	see [1] table 2; Gene re-arrangement and insertion in the PBCV-1_A250R-homolog locus	MA, USA	Aug 1984
NE-JV4	Chlorella NC64A	regular plaques of medium-to-large size	Rowe Bird Sanctuary; NE, USA	May 2008
Chlorella heliozoae SAG 3.83 Virus Isolates				
NTS-1	Chlorella SAG3.83	Alkaline lake isolates, fuzzy plaques	Next to Smith Lake, NE, USA (CLNWR)	June 2008
Canal-1	Chlorella SAG3.83	Alkaline lake isolates, fuzzy plaques; does not completely lyse a culture	canal exiting Smith Lake, NE, USA (CLNWR)	June 2008
TN603.4.2	Chlorella SAG3.83	First SAG 3.83 virus found in the USA; Large, clear plaques;	Tennessee, USA	April 2006
WI0606	Chlorella SAG3.83	Normal plaque size and shape; Geographic site	Madison Wisconsin, USA	July 2006
Br0604L	Chlorella SAG3.83	Normal plaque size and shape; Geographic site	St. Paul, Brazil	2006
GM0701.1	Chlorella SAG3.83	Normal plaque size and shape; Geographic site	Guatemala	January 2007
MO0605SPH	Chlorella SAG3.83	Cloudy plaques; Geographic site	Missouri, USA	2006
Can0610SP	Chlorella SAG3.83	Normal plaque size and shape; Geographic site	British Columbia, Canada	August 2006
OR0704.3	Chlorella SAG3.83	Normal plaque size and shape; Geographic site	Willamette River, Corvallis, Oregon, USA	July 2007
MN0810.1	Chlorella SAG3.83	Normal plaque size and shape; Geographic site (abandoned mine)	Minnesota, USA	August 2008
NE-JV2	Chlorella SAG3.83	small, irregularly shaped plaques with fuzzy edges	Rowe Bird Sanctuary; Gibbon, NE, USA	May 2008
NE-JV3	Chlorella SAG3.83	medium sized, irregularly shaped plaques with fuzzy edges	Gudmundsen Ranch	May 2008

Virus	Host	Attributes and comments (plaque size, plaque morphology, gene content, etc.)	Source of isolate	Date collected
Micractinium conductrix Pbi Virus Isolates				
CVA-1	Micractinium Pbi	see [2]; Normal plaque size and shape	Amönau, Germany	1984
CVB-1	Micractinium Pbi	see [2]; Normal plaque size and shape	Berlin, Germany	1984
CVG-1	Micractinium Pbi	see [2]; Normal plaque size and shape	Göttingen, Germany	1984
CVM-1	Micractinium Pbi	see [2]; Normal plaque size and shape	Marburg, Germany	1984
CVR-1	Micractinium Pbi	see [2]; Normal plaque size and shape	Rauschenberg, Germany	1984
NW665.2	Micractinium Pbi	Small, regularly shaped plaques	Norway	1995
AP110A	Micractinium Pbi	High plaque numbers; 1 of a collection of 10 "AP" viruses	unknown	unknown
Can18-4	Micractinium Pbi	Normal plaque size and shape; Geographic site	Canada	1995
CZ-2	Micractinium Pbi	Normal plaque size and shape; Geographic site	Czech Republic	1995
Fr5L	Micractinium Pbi	Normal plaque size and shape; Geographic site	France	1995
OR0704.2.2	Micractinium Pbi	Normal plaque size and shape; Geographic site	Willamette River, Corvallis, Oregon	July 2007
NE-JV1	Micractinium Pbi	Small, regularly shaped plaques with fuzzy edges	South Platte River, Nebraska	May 2008

1. Van Etten JL, Lane LC, Meints RH: **Viruses and virus-like particles of eukaryotic algae**. *Microbiol. Rev.* 1991, **55**:586–620.

2. Reisser W, Burbank D, Meints R, Becker B, Van Etten J: **Viruses distinguish symbiotic Chlorella spp. of Paramecium bursaria**. *Endocytobiosis and Cell Research* 1991, **7**:247–251.
